# Effect of High vs Low Doses of Chloroquine Diphosphate as Adjunctive Therapy for Patients Hospitalized With Severe Acute Respiratory Syndrome Coronavirus 2 (SARS-CoV-2) Infection: A Randomized Clinical Trial

**DOI:** 10.1001/jamanetworkopen.2020.8857

**Published:** 2020-04-24

**Authors:** Mayla Gabriela Silva Borba, Fernando Fonseca Almeida Val, Vanderson Souza Sampaio, Marcia Almeida Araújo Alexandre, Gisely Cardoso Melo, Marcelo Brito, Maria Paula Gomes Mourão, José Diego Brito-Sousa, Djane Baía-da-Silva, Marcus Vinitius Farias Guerra, Ludhmila Abrahão Hajjar, Rosemary Costa Pinto, Antonio Alcirley Silva Balieiro, Antônio Guilherme Fonseca Pacheco, James Dean Oliveira Santos, Felipe Gomes Naveca, Mariana Simão Xavier, André Machado Siqueira, Alexandre Schwarzbold, Júlio Croda, Maurício Lacerda Nogueira, Gustavo Adolfo Sierra Romero, Quique Bassat, Cor Jesus Fontes, Bernardino Cláudio Albuquerque, Cláudio-Tadeu Daniel-Ribeiro, Wuelton Marcelo Monteiro, Marcus Vinícius Guimarães Lacerda

**Affiliations:** 1 Fundação de Medicina Tropical Dr Heitor Vieira Dourado, Manaus, Brazil; 2 Universidade do Estado do Amazonas, Manaus, Brazil; 3 Fundação de Vigilância em Saúde do Amazonas, Manaus, Brazil; 4 Faculdade de Medicina da Universidade de São Paulo, São Paulo, Brazil; 5 Instituto Leônidas and Maria Deane, Fiocruz Amazonas, Manaus, Brazil; 6 Programa de Computação Científica, Fiocruz, Rio de Janeiro, Brazil; 7 Universidade Federal do Amazonas, Manaus, Brazil; 8 Instituto Nacional de Infectologia Carlos Chagas–Fiocruz, Rio de Janeiro, Brazil; 9 Universidade Federal de Santa Maria, Rio Grande do Sul, Brazil; 10 Faculdade de Medicina da Universidade Federal do Mato Grosso do Sul, Campo Grande, Brazil; 11 Fundação Oswaldo Cruz, Mato Grosso do Sul, Campo Grande, Brazil; 12 Faculdade de Medicina de São José do Rio Preto, São Paulo, Brazil; 13 Universidade de Brasília, Brasília, Brazil; 14 ISGlobal, Hospital Clínic–Universitat de Barcelona, Barcelona, Spain; 15 Centro de Investigação em Saúde de Manhiça, Maputo, Mozambique; 16 Institució Catalana de Recerca i Estudis Avançats (ICREA), Barcelona, Spain; 17 Pediatric Infectious Diseases Unit, Pediatrics Department, Hospital Sant Joan de Déu, University of Barcelona, Barcelona, Spain; 18 Consorcio de Investigación Biomédica en Red de Epidemiología y Salud Pública, Madrid, Spain; 19 Universidade Federal de Mato Grosso, Mato Grosso, Brazil; 20 Faculdade de Medicina da Universidade Federal do Amazonas, Manaus, Brazil; 21 Instituto Oswaldo Cruz, Fundação Oswaldo Cruz, Rio de Janeiro, Brazil

## Abstract

**Importance:**

There is no specific antiviral therapy recommended for coronavirus disease 2019 (COVID-19). In vitro studies indicate that the antiviral effect of chloroquine diphosphate (CQ) requires a high concentration of the drug.

**Objective:**

To evaluate the safety and efficacy of 2 CQ dosages in patients with severe COVID-19.

**Design, Setting, and Participants:**

This parallel, double-masked, randomized, phase IIb clinical trial with 81 adult patients who were hospitalized with severe acute respiratory syndrome coronavirus 2 (SARS-CoV-2) infection was conducted from March 23 to April 5, 2020, at a tertiary care facility in Manaus, Brazilian Amazon.

**Interventions:**

Patients were allocated to receive high-dosage CQ (ie, 600 mg CQ twice daily for 10 days) or low-dosage CQ (ie, 450 mg twice daily on day 1 and once daily for 4 days).

**Main Outcomes and Measures:**

Primary outcome was reduction in lethality by at least 50% in the high-dosage group compared with the low-dosage group. Data presented here refer primarily to safety and lethality outcomes during treatment on day 13. Secondary end points included participant clinical status, laboratory examinations, and electrocardiogram results. Outcomes will be presented to day 28. Viral respiratory secretion RNA detection was performed on days 0 and 4.

**Results:**

Out of a predefined sample size of 440 patients, 81 were enrolled (41 [50.6%] to high-dosage group and 40 [49.4%] to low-dosage group). Enrolled patients had a mean (SD) age of 51.1 (13.9) years, and most (60 [75.3%]) were men. Older age (mean [SD] age, 54.7 [13.7] years vs 47.4 [13.3] years) and more heart disease (5 of 28 [17.9%] vs 0) were seen in the high-dose group. Viral RNA was detected in 31 of 40 (77.5%) and 31 of 41 (75.6%) patients in the low-dosage and high-dosage groups, respectively. Lethality until day 13 was 39.0% in the high-dosage group (16 of 41) and 15.0% in the low-dosage group (6 of 40). The high-dosage group presented more instance of QTc interval greater than 500 milliseconds (7 of 37 [18.9%]) compared with the low-dosage group (4 of 36 [11.1%]). Respiratory secretion at day 4 was negative in only 6 of 27 patients (22.2%).

**Conclusions and Relevance:**

The preliminary findings of this study suggest that the higher CQ dosage should not be recommended for critically ill patients with COVID-19 because of its potential safety hazards, especially when taken concurrently with azithromycin and oseltamivir. These findings cannot be extrapolated to patients with nonsevere COVID-19.

**Trial Registration:**

ClinicalTrials.gov Identifier: NCT04323527

## Key Points

**Question:**

How safe and effective are 2 different regimens of chloroquine diphosphate in the treatment of severe coronavirus disease 2019 (COVID-19)?

**Findings:**

In this phase IIb randomized clinical trial of 81 patients with COVID-19, an unplanned interim analysis recommended by an independent data safety and monitoring board found that a higher dosage of chloroquine diphosphate for 10 days was associated with more toxic effects and lethality, particularly affecting QTc interval prolongation. The limited sample size did not allow the study to show any benefit overall regarding treatment efficacy.

**Meaning:**

The preliminary findings from the CloroCovid-19 trial suggest that higher dosage of chloroquine should not be recommended for the treatment of severe COVID-19, especially among patients also receiving azithromycin and oseltamivir, because of safety concerns regarding QTc interval prolongation and increased lethality.

## Teaser

This randomized clinical trial evaluates the safety and efficacy of 2 chloroquine diphosphate dosages in patients with severe coronavirus disease 2019 (COVID-19).

## Introduction

The first cases of the new coronavirus 2019 disease (COVID-19) were reported in December 2019 when a group of patients was admitted to hospitals in Wuhan, the capital of Hubei province in central China, with an initial diagnosis of pneumonia of unknown etiology.^1^ Initially, the outbreak of severe acute respiratory syndrome coronavirus 2 (SARS-CoV-2) was confined to Hubei province, but it rapidly spread to many other countries,^2,3^ compelling the World Health Organization to officially declare a global pandemic on March 11, 2020. SARS-CoV-2 infection appears to cause a wide range of symptoms.^4,5^ Most deaths involve older adults, many of whom had underlying chronic diseases.^6,7^

Recent publications have drawn attention to the possible benefit of chloroquine diphosphate (CQ) and hydroxychloroquine (HCQ) for the treatment of patients with SARS-CoV-2 infection.^8,9,10,11,12,13^ Both drugs have been used for the treatment of acute malaria as well as for some chronic rheumatic conditions. Hydroxychloroquine, a derivative of CQ first synthesized in 1946, proved to be less toxic (by approximately 40%) when used for longer periods and has been recommended for the treatment of systemic lupus erythematosus and rheumatoid arthritis.^14^ During prolonged use (ie, months or even years), which is not the targeted scenario for the treatment of COVID-19, CQ may deposit in the eye, causing retinal toxicity.^15,16^ Myopathy has also been associated with the use of CQ.^17^ The major complication, even in short regimens, is the potential for QTc interval prolongation, favoring fatal arrhythmias such as ventricular tachycardia and torsades de pointes.^18^ The in vitro antiviral activity of CQ was first identified in the late 1960s.^19,20^ Two studies have shown anti–SARS-CoV activity, with high concentrations needed for antiviral effect.^9,11^

The effect of CQ was apparently superior to the control treatment in inhibiting the exacerbation of pneumonia, improving pulmonary imaging findings, promoting a negative conversion of the virus, and reducing the disease course.^12^ In 20 patients with COVID-19 treated with HCQ, 6 of whom also received azithromycin, the proportion of patients who tested negative in nasopharyngeal samples differed significantly between patients receiving treatment and patients in the control group.^13^ Although highly preliminary and probably not sufficiently powered to be conclusive, these results supported an effort to evaluate the effect of CQ on the evolution and prognosis of COVID-19 more thoroughly.

The Health Commission of Guangdong Province recommended the use of phosphate CQ tablets at a dose of 500 mg twice daily for 10 days (total dose, 10 g) for the treatment of patients aged 18 to 65 years with mild, moderate, or severe pneumonia secondary to COVID-19.^10^ A shorter treatment regimen (ie, 5 vs 10 days) could potentially reduce the adverse effects, but the antiviral effect could be lost. Therefore, no clear recommendation of total dosage is available; most recommendations are based on expert opinion.

Considering that in many countries the compassionate use of CQ or HCQ to treat COVID-19 has already been formally indicated for patients with severe disease, it would be unethical to test proper efficacy owing to the lack of a placebo group as a comparator. Our study aimed to evaluate primarily the safety and secondarily the efficacy of CQ in 2 different dosages for the treatment of severe COVID-19. Here, we report data from the first 81 randomized patients after an unplanned interim analysis due to safety concerns recommended by the independent data safety and monitoring board (DSMB).

## Methods

The detailed study protocol is available in Supplement 1. This study was conducted in accordance with the principles of the Declaration of Helsinki and the Good Clinical Practice guidelines of the International Conference on Harmonization. The protocol was approved by the Brazilian Committee of Ethics in Human Research. All patients and/or legal representatives were informed about objectives and risks of participation. They were given time to carefully read and then sign an informed consent form. After recovery, patients also signed the informed consent form. Random online clinical monitoring and quality control were performed. A virtual independent DSMB, composed of epidemiologists, clinicians, and experts in infectious diseases, was implemented to review the protocol and hold daily meetings to follow the activities of the study. The trial was reported according to the Consolidated Standards of Reporting Trials (CONSORT) reporting guideline.^21^

### Study Design and Site

CloroCovid-19 was a parallel, double-masked, randomized, phase IIb clinical trial, which started on March 23, 2020, aiming to assess the safety and efficacy of CQ in the treatment of hospitalized patients with severe respiratory syndrome secondary to SARS-CoV-2 infection. This trial is being conducted at the Hospital e Pronto-Socorro Delphina Rinaldi Abdel Aziz, in Manaus, Western Brazilian Amazon (currently the largest public unit dedicated exclusively to the treatment of severe COVID-19 cases in Brazil, with the capacity to hospitalize 350 patients in intensive care units). The hospital has all source documents registered online in an electronic medical recording system (Medview). Clinical analyses, laboratory examinations, and routine computed tomography scanning are also available locally. Manaus is the capital of the Amazonas state, the largest Brazilian state, and has approximately 2.5 million inhabitants. At the beginning of the study, autochthonous SARS-CoV-2 transmission had already been recorded at the study site.

## Participants

**Figure 1.**
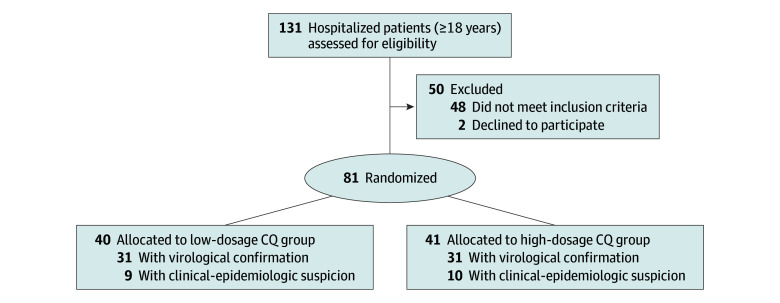
Study Flow Chart Eligible participants were allocated at a 1:1 ratio to receive chloroquine (CQ) in 2 groups at either high dosage (600 mg CQ twice daily for 10 days) or low dosage (450 mg CQ twice daily on the first day and 450 mg once daily for 4 days).

Hospitalized patients with clinical suspicion of COVID-19 (ie, history of fever and any respiratory symptom, eg, cough or rhinorrhea), aged 18 years or older at the time of inclusion, with respiratory rate higher than 24 rpm and/or heart rate higher than 125 bpm (in the absence of fever) and/or peripheral oxygen saturation lower than 90% in ambient air and/or shock (ie, arterial pressure lower than 65 mm Hg, with the need for vasopressor medicines, oliguria, or a lower level of consciousness) were included. Patients younger than 18 years were not included due to the known lower morbidity and mortality from COVID-19 in this group.^22^ Patients were enrolled before laboratory confirmation of COVID-19, considering that this procedure could delay randomization. For the analyses at this point, all patients were included regardless of confirmed etiology, which should not be an issue for the focus of this article, ie, safety concerns. The flow chart (Figure 1) presents clinical-epidemiologic suspected cases as well as cases already confirmed by reverse transcription–polymerase chain reaction.

### Sample Size Calculation

The sample for the primary outcome (ie, reduction in lethality rate) was calculated assuming a 20% lethality incidence in critically ill patients^7,23,24^ and that higher dose of CQ would reduce lethality by at least 50% compared with the low-dosage group. Thus, considering a test of differences in proportions between 2 groups of the same size, 80% power and 5% α, 394 participants were needed (197 per group). Adding 10% for losses, the final sample of 440 participants was obtained. Sample calculation was performed in the R version 3.6.1 (R Project for Statistical Computing), with the functions implemented in the *TrialSize* and *gsDesign* packages.

### Procedures

Dosages in the literature are heterogeneous. The reasons that guided the high dosage in this study were as follows: (1) in principle, antiviral dosages should be high, as per in vitro studies’ results^9^; (2) the toxic effects of high doses, such as HCQ 600 mg twice daily for 28 days, were already studied in patients with cancer, showing good safety even in phase I trials^25,26,27^; (3) in an unknown disease that has proved to be more lethal than expected, the benefits for critically ill patients were thought to be superior to the adverse effects of high-dose CQ; (4) only 150 mg chloroquine base tablets are available in Brazil, which needed to be adjusted for a routine daily prescription to avoid tablet partition; (5) patients with high body mass index (calculated as weight in kilograms divided by height in meters squared) in the study population (ie, median [interquartile range], 28.1 [26.0-31.6]); and (6) critically ill patients in shock usually present limited gastrointestinal absorption, and no intravenous drug was available. The low dosage was what was recommended by the Brazilian Ministry of Health, based on expert opinion.

The interventions tested in this study were based on different regimens using CQ base 150 mg tablets (241.9 mg of the phosphate CQ per tablet) (Farmanguinhos). Eligible participants were allocated at a 1:1 ratio to receive orally (or via nasogastric tube in case of orotracheal intubation) either high-dosage CQ (600 mg CQ; 4 × 150 mg tablets twice daily for 10 days; total dose 12 g) or low-dosage CQ (450 mg CQ; 3 × 150 mg tablets and 1 placebo tablet twice daily on day 0, 3 × 150 mg tablets plus 1 placebo tablet once a day followed by 4 placebo tablets from day 1 to day 4, then 4 placebo tablets twice daily from day 5 to day 9; total dose 2.7 g). Placebo tablets, also produced by Farmanguinhos, were used in the low-dosage group to standardize treatment and masking of research team and participants.

According to hospital protocol, all patients meeting the same criteria of the study (ie, acute respiratory distress syndrome) received intravenous ceftriaxone (1 g twice daily for 7 days) plus azithromycin (500 mg once daily for 5 days), systematically, starting on day 0. Oseltamivir (75 mg twice daily for 5 days) was also prescribed when influenza infection was suspected. (In the Amazon, influenza season is from January to April.)

Clinical parameters were measured daily by the routine clinical staff from day 0 to discharge or death, and then at days 13 and 28 for discharged patients, to assess efficacy and safety outcomes. Laboratory parameters and electrocardiograms were performed at the clinician’s discretion. Data were recorded on Medview and then transferred into an electronic database (Research Electronic Data Capture) on tablet computers at bedside in the wards, which were further validated by external trial monitoring staff.

### Outcomes

Safety outcomes included adverse events that occurred during treatment, serious adverse events, and premature or temporary discontinuation of treatment. Adverse events were classified according to the National Cancer Institute Common Terminology Criteria for Adverse Events. The working hypothesis of this trial was that the lethality rate in the high-dosage group would be half that of the low-dosage group by day 28. Thus, the primary end point was lethality by day 28. Secondary end points included lethality on day 13, participant clinical status, laboratory examinations, electrocardiogram on days 13 and 28, daily clinical status during hospitalization, duration of mechanical ventilation (if applicable) and supplementary oxygen (if applicable), and the time (in days) from treatment initiation to death. Here we present analyses until day 13, with lethality as the primary outcome. A subgroup of patients enrolled when already admitted to the intensive care unit was analyzed separately. Virologic measures included viral RNA detection on days 0 and 4.

### Randomization and Masking

An electronically generated randomization list was prepared by an independent statistician, with 110 blocks of 4 participants per block. This randomization list was generated on R version 3.6.3 (R Project for Statistical Computing), using the package *blockrand*. The list was accessible only to nonmasked pharmacists in the study in an attempt to minimize observation bias. Participants were randomized by the study pharmacist to their designated treatment regimen at the time of inclusion and were subsequently identified throughout the study only by their allocated study number, always assigned in chronological order. In case of serious adverse events, unmasking was available to DSMB members, and an unplanned preliminary analysis was performed before the scheduled interim analyses to guide early halting of either group. At this point, an overall lethality rate higher than 25% was noted, and serious cardiac adverse events were reported.

### Laboratory Analysis

Hematology and biochemistry analyses were performed in automatized machines. Samples (2 nasopharyngeal or 1 oropharyngeal swabs) were submitted to viral RNA extraction using QIAamp Viral RNA Mini Kit (Qiagen) according to the manufacturer’s recommendations. Subsequently, all specimens of potential SARS-CoV-2 were tested using the protocol developed by the US Centers for Disease Control and Prevention, updated on March 15, 2020,^28^ targeting the virus nucleocapsid (N) gene and the human RNase P gene as an internal control. For all assays, specimens were considered positive if both viral targets (ie, N1 and N2) showed cycle threshold lower than 40.00. No quantitative reverse transcription–polymerase chain reaction data are presented here. Swab specimens were collected on day 0 and day 4. Results were not available to guide any clinical decision because a state-level laboratory (LACEN) centralized the examinations.

### Statistical Analysis

We originally planned to perform an interim analysis between the groups when the study reached 25%, 50%, and 75% of the total sample size. However, global lethality (without unmasking) was measured daily for security purposes, and the DSMB was informed accordingly. An intention-to-treat analysis was conducted as part of the primary safety and efficacy analysis. Untaken or mistaken tablets and dosage correction because of renal and liver failure were not registered daily, therefore not allowing for per-protocol analysis. Descriptive statistics were used for demographic, laboratory, and clinical data. To assess the safety of the high and low dosages of CQ, the proportion (and 95% CI) of deaths in each group was compared with the historical proportion (and 95% CI) of deaths in patients who did not use CQ in other countries.^7,23,24^ For qualitative variables, χ^2^ tests and Fisher exact tests were performed. We used the *t* test or Mann-Whitney test to compare means and medians. Survival models, using Kaplan-Meier estimate curves, assessed the cumulative proportion of deaths. Log-rank and Peto-Peto (correction for low observation numbers in the end of the follow-up) tests were used for survival time to event analyses. Exploratory multivariate analysis was performed using logistic regression to assess the strength of the association between treatment arm and lethality, adjusted by age. Odds ratios with respective 95% CIs were calculated. Statistical analyses were performed in R version 3.6.1 (R Project for Statistical Computing), and a 2-tailed *P* < .05 was considered significant.

## Results

### Population Characteristics

A total of 81 patients were randomized (40 [49.4%] in the low-dosage group and 41 [50.6%] in the high-dosage group) (Figure 1). A preliminary analysis was performed on April 5, 2020, per DSMB recommendation, when 11 patients had died (7 [63.6%] in the high-dosage group; 4 [36.4%] in the low-dosage group). Most patients (62 of 81 [76.5%]) had COVID-19 confirmed a posteriori by reverse transcription–polymerase chain reaction, with 31 (77.5%) in the low-dosage group and 31 (75.6%) in the high-dosage group. The patients with unconfirmed disease had clinical and epidemiological presentation compatible with COVID-19 and were analyzed together.

Overall and per-group baseline characteristics are presented in Table 1. Baseline characteristics show an overall mean (SD) age of 51.1 (13.9) years and a predominance of men (60 [75.3%]). Hypertension (25 of 55 [45.5%]), alcohol use disorder (14 of 51 [27.5%]), and diabetes (14 of 55 [25.5%]) were the most frequent comorbidities. Older age (mean [SD] age, 54.7 [13.7] years vs 47.4 [13.3] years) and more heart disease (5 of 28 [17.9%] vs 0) were seen in the high-dose group

**Table 1.** Demographic, Clinical, Laboratory, and Radiographic Findings of Patients at Baseline

Variable	No./total No. (%)^a^		
	Overall cohort (N = 81)	Low-dosage group (n = 40)^b^	High-dosage group (n = 41)^c^
Age, mean (SD), y	51.1 (13.9)	47.4 (13.3)	54.7 (13.7)
Women	20/81 (24.7)	10/40 (25.0)	10/41 (24.4)
Race			
White	17/81 (21.0)	10/40 (25.0)	7/41 (17.1)
Mixed	58/81 (71.6)	28/40 (70.0)	30/41 (73.2)
Black	6/81 (7.4)	2/40 (5.0)	4/41 (9.8)
Pregnant	2/20 (10.0)	1/10 (10.0)	1/10 (10.0)
History of smoking			
Never	33/48 (68.8)	18/24 (75.0)	15/24 (62.5)
Current	4/48 (8.3)	3/24 (12.5)	1/24 (4.2)
Former	11/48 (22.9)	3/24 (12.5)	8/24 (33.3)
Comorbidities			
Hypertension	25/55 (45.5)	10/27 (37)	15/28 (53.6)
Diabetes	14/55 (25.5)	5/27 (18.5)	9/28 (32.1)
Alcohol use disorder	14/51 (27.5)	8/26 (30.8)	6/25 (24)
Heart disease	5/55 (9.1)	0/27	5/28 (17.9)
Asthma	4/54 (7.4)	1/26 (3.8)	3/28 (10.7)
Chronic kidney disease	4/54 (7.4)	1/26 (3.8)	3/28 (10.7)
Rheumatic diseases	3/55 (5.5)	3/27 (11.1)	0/28
Liver diseases	2/55 (3.6)	2/27 (7.4)	0/28
Tuberculosis	2/55 (3.6)	2/27 (7.4)	0/28
HIV/AIDS	1/55 (1.8)	0/27	1/28 (3.6)
Oxygen therapy on admission	72/81 (88.9)	36/40 (90.0)	36/41 (87.8)
Body temperature, °C			
<37.5	59/79 (74.7)	30/39 (76.9)	29/40 (72.5)
37.5-38.0	10/79 (12.7)	6/39 (15.4)	4/40 (10)
38.1-39.0	10/79 (12.7)	3/39 (7.7)	7/40 (17.5)
Heart rate, mean (SD), bpm	91 (17.5)	91.6 (18.7)	90.4 (16.4)
Respiratory rate, median (IQR), rpm	26.0 (21.0-30.0)	25.0 (22.0-30.0)	28.0 (20.0-31.0)
Mean blood pressure, mean (SD), mm Hg	94.4 (17.1)	96.2 (18.8)	92.7 (15.4)
BMI, median (IQR)	28.1 (26.0-31.6)	28.9 (26.1-32.7)	27.1 (25.7-31.2)
Capillary refill time, sec	13/55 (23.6)	6/26 (23.07)	7/27 (25.9)
Oxygen saturation, median (IQR), %	96 (94.0-98.0)	96 (93.0-98.0)	95 (94.0-98.2)
White blood cell count, mean (SD), /μL	10 100 (4600)	10 000 (4500)	10 200 (4800)
Hemoglobin, mean (SD), g/dL	1.28 (0.23)	1.32 (0.26)	1.24 (0.19)
Platelet count, median (IQR), ×10^3^/μL	211.0 (182.8-258.5)	196.5 (172.5-256)	215.0 (184.2-257.5)
Alanine aminotransferase, median (IQR), U/L	65.2 (49.7-103.8)	51 (39.1-53.8)	100 (92.3-115.1)
Creatinine, median (IQR), mg/dL	0.02 (0.01-0.03)	0.01 (0.01-0.02)	0.02 (0.01-0.03)
Lactate dehydrogenase, median (IQR), U/L	948 (810.0-1139.8)	900 (553.0-1009.0)	1010 (869.0-1337.5)
Creatine, median (IQR), U/L			
Kinase	95.2 (61.9-250.4)	82.8 (55.8-177.4)	96.8 (70.8-279.0)
Kinase MB	20 (15.8-25.9)	18.6 (15.8-24.5)	20.9 (15.8-27.3)
C-reactive protein, median (IQR), mg/dL	8.48 (6.98-9.47)	8.09 (6.19-9.51)	8.61 (7.73-9.19)
QTc interval, mean (SD), milliseconds	424.7 (27.4)	421.9 (24.0)	427.8 (31.0)
Radiologic findings			
Ground-glass opacity infiltration			
Unilateral	41/81 (50.6)	20/40 (50.0)	21/41 (51.2)
Bilateral	8/81 (9.9)	6/40 (15.0)	2/41 (4.9)
Consolidation			
Unilateral	25/81 (30.9)	15/40 (37.5)	10/41 (24.4)
Bilateral	15/81 (18.5)	7/40 (17.5)	8/41 (19.5)
Pleural effusion	5/81 (6.2)	3/40 (7.5)	2/41 (4.9)
qSOFA score ≥2	27/81 (33.3)	10/40 (25.0)	17/41 (41.5)

Abbreviations: BMI, body mass index (calculated as weight in kilograms divided by height in meters squared); IQR, interquartile range; qSOFA, quick sequential organ failure assessment.

SI conversion factors: To convert alanine aminotransferase to microkatals per liter, multiply by 0.0167; C-reactive protein to milligrams per liter, multiply by 10.0; creatine kinase to microkatals per liter, multiply by 0.0167; creatinine to micromoles per liter, multiply by 88.4; hemoglobin to grams per liter, multiply by 10.0; lactate dehydrogenase to microkatal per liter, multiply by 0.167; platelet count to ×10^9^ per liter, multiply by 1.0; and white blood cell count to ×10^9^ per liter, multiply by 0.001.

^a^ For some variables, patients’ unconsciousness did not allow for complete personal history data collection.

^b^ Low-dosage group received chloroquine for 5 days (450 mg twice daily on the first day and 450 mg once daily for 4 days).

^c^ High-dosage group received chloroquine for 10 days (600 mg twice daily for 10 days).

Occurrence of myocarditis (defined as a creatine kinase-MB [CKMB] level more than twice the upper normal limit), which may be a final complication of severe sepsis or a lesion triggered by the virus itself, was seen in 2 of 26 (7.7%) patients (1 patient per group). No echocardiogram was performed. All patients received azithromycin, and the frequency of oseltamivir use was 86.8% (33 of 38) and 92.5% (37 of 40) in the low- and high-dosage groups, respectively.

### Safety Outcomes

Creatine phosphokinase (CK) and CKMB levels were elevated in 13 of 33 patients (39.4%) and 10 of 26 patients (38.4%), respectively. Considering only confirmed COVID-19 cases, CK and CKMB were elevated in 9 of 25 patients (37.5%) and 7 of 22 patients (31.8%), respectively, and CK increase was more frequent in patients in the high-dosage group than the low-dosage group (7 of 14 [50.0%] vs 6 of 19 [31.6%]). Only 1 patient developed severe rhabdomyolysis, and causality could be attributed to the virus or to CQ, which is already known to cause myolysis (Table 2). Overall 11 of 73 patients (15.1%) had QTc interval corrected by the Fridericia method (QTcF) greater than 500 milliseconds, with 8 of 57 patients (14.0%) with confirmed cases of COVID-19. QTcF greater than 500 milliseconds was more frequent in the high-dosage group than the low-dosage group (7 of 37 [18.9%] vs 4 of 36 [11.1%]). Two of 37 patients (2.7%) in the high-dosage group, both with confirmed COVID-19, experienced ventricular tachycardia before death, without torsade de pointes. This severe type of arrythmia is usually facilitated when QTc interval is prolonged. We did not calculate CQ dose by weight; however, only 1 patient (1.2%) weighed less than 110 lbs. Body mass index was similar in both groups.

**Table 2.** Safety Outcomes in the Intention-to-Treat Population Until Day 13^a^

Variable	No/ total No. (%)					
	All patients			COVID-19 confirmed cases		
	Total	Low-dosage group^b^	High-dosage group^c^	Total	Low-dosage group^b^	High-dosage group^c^
Hemoglobin decreased^d^	11/42 (26.2)	4/18 (22.2)	7/24 (19.2)	7/29 (24.1)	3/11 (27.3)	4/18 (22.2)
Creatinine increased^e^	16/38 (42.1)	7/15 (46.7)	9/23 (39.1)	13/27 (48.1)	5/9 (55.6)	8/18 (44.4)
CK increased	13/33 (39.4)	6/19 (31.6)	7/14 (50.0)	9/24 (37.5)	3/15 (20.0)	6/9 (66.7)
CKMB increased	10/26 (38.4)	3/13 (23.1)	7/13 (53.8)	7/22 (31.8)	3/13 (23.1)	4/9 (44.4)
QTcF >500 ms^f^	11/73 (15.1)	4/36 (11.1)	7/37 (18.9)	8/57 (14.0)	1/27 (3.6)	7/29 (24.1)
Ventricular tachycardia	2/73 (2.7)	0/36	2/37 (2.7)	2/62 (3.2)	0/31	2/31 (6.5)

Abbreviation: CK, creatine phosphokinase; CKMB, creatinine phosphokinase–MB; COVID-19, coronavirus disease 2019; QTcF, QT interval corrected by the Fridericia method.

^a^ Not all patients completed day 13 visit before this article was finalized.

^b^ Low-dosage group received chloroquine for 5 days (450 mg twice daily on the first day and 450 mg once daily for 4 days).

^c^ High-dosage group received chloroquine for 10 days (600 mg twice daily for 10 days).

^d^ Decreases in hemoglobin level of more than 3 g/dL or 30% or greater from baseline are shown.

^e^ Increases in creatinine serum levels of 30% or more from baseline are shown.

^f^ Serious adverse events related to the trial regimen were prolongation of the QTcF.

Hemoglobin decrease was observed in 11 of 42 patients (26.2%). Creatinine increase was observed in 16 of 38 (42.1%). No apparent differences in hematological or renal toxicity were seen between the groups.

### Lethality Outcomes

**Figure 2.**
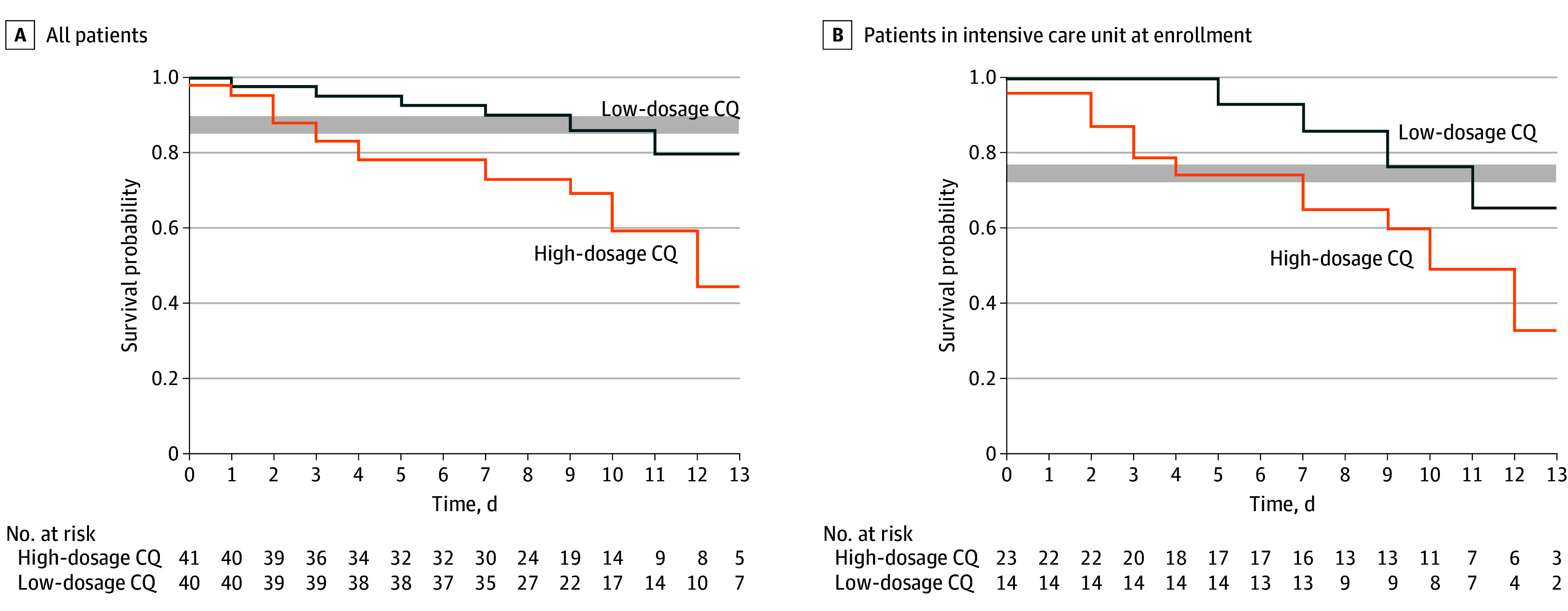
Time From Randomization to Death Among Patients Treated With Each Chloroquine (CQ) Dosage A, The gray band represents the upper and lower limits of the confidence interval for lethality in hospitalized patients not receiving CQ obtained by Zhou et al^7^ and Chen et al^23^ (ie, 167 of 990 patients [16.9%]; 95% CI, 14.5%-19.2%). B, The gray band represents the upper and lower limits of the confidence interval for lethality in critically ill patients not receiving CQ in the study by Grasselli et al^24^ (ie, 405 of 1581 [25.6%]; 95% CI, 23.5%-27.8%).

Overall lethality rate in our sample was 27.2% (95% CI, 17.9%-38.2%), which overlapped with the 95% CI of the meta-analysis^7,23^ based on 2 major studies (95% CI, 14.5%-19.2%) that included similar patients not receiving CQ. Survival per group is presented in comparison with historical collation of available data from 2 other similar lethality studies^7,23^ with patients not receiving CQ (Figure 2A). Lethality was 39.0% (16 of 41 patients) in the high-dosage group and 15.0% (6 of 40) in the low-dosage group. Survival analysis has shown that both groups were similar to historical data, showing no apparent differences despite more deaths in the high-dosage group (log-rank, −2.183; *P* = .03). A similar survival analysis excluding 5 patients (6.2%) with chronic cardiac disease was performed, and similar results were found (log-rank, −2.188; *P* = .03). Viral RNA was detected in 5 of 6 (83.3%) and 14 of 16 (87.5%) of the dead patients in the low-dosage and high-dosage groups, respectively.

The high-dosage group was associated with lethality (odds ratio, 3.6; 95% CI, 1.2-10.6). Despite the small sample size, in an exploratory multivariate analysis, the high-dosage CQ was no longer associated with death when controlled by age (odds ratio, 2.8; 95% CI, 0.9-8.5). In 5 patients with chronic cardiac disease, 3 (60.0%) died; their clinical details are presented in Table 3. Neither ventricular tachycardia nor torsade de pointes was seen in these patients. In the eTable in Supplement 2, we present details of 12 patients with prolonged QTcF and/or ventricular tachycardia. No clear association was seen between the first day of prolonged QTcF and day of death, nor did cumulative dosages seem to be higher among those who died. Overall, 19 of 22 deaths (86.4%) had virologic confirmation of SARS-CoV-2 infection antemortem. Based on these findings, in which a higher dosage of CQ showed the opposite of the study’s hypothesis, the DSMB recommended the immediate interruption of the high-dosage group for all ages and that all patients be unmasked and reverted to the low-dosage group.

**Table 3.** Clinical Details of 5 Patients Enrolled in the High-Dosage Group With Previous Cardiac Disease

Age, y	Sex	Race	First QTcF, ms	Previous cardiac disease	Other comorbidities	Death
70s	Woman	Black	478	Heart failure	Hypertension, diabetes, and chronic kidney disease	No
60s	Man	Mixed	488	Coronary chronic disease	Hypertension and diabetes	Yes
40s	Woman	White	457	Heart failure	Chronic obstructive pulmonary disease and obesity	No
60s	Man	Mixed	440	Coronary chronic disease	None	Yes
70s	Man	White	NA	Atrioventricular block	Hypertension	Yes

Abbreviations: NA, not available; QTcF, QT interval corrected by the Fridericia method.

A subgroup was analyzed with critically ill patients enrolled (Figure 2B). No difference in lethality rates was seen between groups.

A total of 27 patients had nasopharyngeal and/or oropharyngeal samples collected on days 0 and 4. Results were negative on day 4 in only 6 patients (22.2%).

## Discussion

In a unique pandemic situation, health professionals have to choose between offering medical assistance and generating and reporting reliable data, a dichotomy that compromises the ability to generate high-quality evidence for clinical management. However, global recommendations for COVID-19 are being made based on unpowered studies, and because of the chaotic urgency of the situation, drugs are being prescribed in a compassionate manner given the severity of the disease. CQ is a safe drug, used for more than 70 years to treat malaria. However, in the context of patients with severe COVID-19, our study raises enough red flags to stop the use of a high-dosage regimen (ie, 12 g of CQ during 10 days), because the risks of toxic effects overcame the benefits.

We were not able to independently assess the toxic role of CQ because all patients were already using azithromycin, as per hospital protocol. Official recommendations from China^10^ called attention to the nonsimultaneous use of CQ and azithromycin because of potentially synergistic cardiac toxic effects. This combination was also used to treat some patients from Marseille, France, without any concerning safety report.^13^ Most patients (89.6%) in our study were also receiving oseltamivir for suspected influenza infection, which also increases QTc interval and could have adverse cardiac effects. The data presented here refer to patients in whom CQ, azithromycin, and oseltamivir were concomitantly used because of the atypical circumstances of an unknown disease. Further conclusions on synergistic cardiotoxic effects might be possible with ongoing studies’ results worldwide, as soon as they are presented to the scientific community.

By the time of the study planning, the Brazilian regulatory agency and the Brazilian Ministry of Health authorized the compassionate use of CQ and HCQ at the clinician’s discretion, with pressure on physicians to prescribe the drug for patients with severe COVID-19. Although this is not an imperative against running placebo-controlled trials, it triggered an ethical dilemma regarding the conduct of randomized clinical trials offering placebo treatment for patients, strongly influenced by the media favoring CQ use. We also accounted for the fact that the standard of care for severe COVID-19 included CQ in the clinical setting where the trial would be conducted. In the absence of a placebo group, we were compelled to use historical data based on very similar patients not receiving CQ. The lethality rates observed here were not lower; however, we cannot reliably conclude that CQ was of no benefit. Placebo-controlled studies are being performed in countries not routinely using the drug.^29^ Several ongoing trials (including the CloroCovid-19 II trial^30^) have also been addressing the early use of CQ, in which the anti-inflammatory properties could potentially be more helpful. That information is urgently needed in well-designed placebo-controlled double-masked randomized trials.

We will still enroll patients in the low-dosage group to complete the originally planned sample size. The need for careful follow-up and toxic effect monitoring of patients using the low-dosage regimen in a scenario where CQ is routinely prescribed for severe COVID-19 cases supported this decision. The safety data obtained from the low-dosage group would be extremely useful for designing better guidelines for the rational use of CQ as compassionate treatment for severe COVID-19 until the conclusion of placebo-controlled trials. All the patients remaining in the study were asked to provide updated informed consent, and the informed consent form was properly modified.

In addition to helping patients improve, CQ could be used to decrease the viral load in respiratory secretions, allowing less nosocomial and postdischarge transmission. However, our data provided no evidence of such an effect. Patients using CQ (irrespective of dosage) failed to present evidence of substantial viral clearance by day 4, even with the concomitant use of azithromycin.

No data exist in the literature showing different cardiotoxic effects between CQ and HCQ; the only concern is with ocular assessment. QTc interval prolongation greater than 500 milliseconds was seen in 11 of 73 patients (15.1%), which is similar to what has been reported in patients with COVID-19 receiving HCQ (11.0%).^31^ Myopathy has also been associated with CQ use.^17^ In our study, 1 patient developed rhabdomyolysis, which was attributed to CQ, and the drug was withdrawn. In 2 patients, myocarditis was suspected based on CKMB elevation since the first day of hospitalization, suggesting myocarditis related to SARS-CoV-2 itself. In such cases, drugs prolonging QTc interval could lead to severe arrhythmias. Unfortunately, probably because of the low sample size, this study’s randomization assigned more older patients with heart disease to the high-dosage group than the low-dosage group. Therefore, a limitation for the conclusions of the study on lethality per group is that the high-dosage group included more patients susceptible to cardiac complications, with or without CQ treatment. In any case, use of CQ in older patients, especially those with heart disease, should be conducted with caution. In our sample, the decision to enroll all types of patients in a pragmatic design, whatever age or comorbidity, was based on the formerly predicted high lethality among critically ill patients with COVID-19, and an imprecise risk-benefit of CQ was assumed at the time of the protocol design. In view of the results, it is clear that any CQ treatment or protocol design for severe COVID-19 should include previous QTc interval evaluation, close daily monitoring, and dosage modification when needed.

Lethality among critically ill patients in the present study seemed to be even higher than among similar patients not receiving CQ in a large historical sample-size cohort of patients in Lombardy, Italy.^24^ That could reflect the quality of intensive care units in both countries or the possible lack of or deleterious effect of CQ in such patients with COVID-19. The occurrence of myocarditis in our sample, with the confirmed QTcF prolongation, warrants caution regarding this drug’s safety, particularly considering the eventual increase in fatal arrythmias, such as ventricular tachycardia.

### Strengths and Limitations

This study had some strengths. It was double-masked; performed in a public hospital, which will manage most cases in countries like Brazil; compliant with good clinical practices, with a vigilant and highly involved DSMB; and presented an assessment of 2 dosages of CQ for the first time in patients with severe COVID-19. However, this study has limitations, including its small sample size; its single-center design; its lack of a placebo control group; and the absence of exclusion criteria based on the QTc interval at baseline.

Per-protocol analysis was not performed because of the impossibility of monitoring drug administration twice a day at the hospital. Radiologic findings were presented in this article only at the baseline due to the inability to perform careful analyses of available computed tomography scans over time. Radiologic and complete efficacy data will be presented later.

## Conclusions

In this study, a high-dosage of CQ (12 g) given for 10 days concurrently with azithromycin and oseltamivir was not sufficiently safe to warrant continuation of that study group. Age was an important confounder and might be associated with the unfavorable outcomes. We recommend that similar dosages no longer be used for the treatment of severe COVID-19, especially because treatment based on older patients with previous cardiac diseases who are receiving concomitant cardiotoxic drugs should be the rule. No apparent benefit of CQ was seen regarding lethality in our patients so far. To better understand the role of CQ or HCQ in the treatment of COVID-19, we recommend the following next steps: (1) randomized clinical trials evaluating its role as a prophylactic drug and (2) randomized clinical trials evaluating its efficacy against the progression of COVID-19 when administered to patients with mild or moderate disease. Even if we fail to generate good evidence in time to control the current pandemic, the information will affect how we deal with coronavirus outbreaks in the future.

## Notes

Supplement 1.
Trial Protocol

Supplement 2. **eTable. **Clinical Details and Cumulative CQ Dosage per Kilogram in 12 Patients With QTcF Prolongation and Ventricular Tachycardia

Supplement 3.
Data Sharing Statement
