# Supplementary material for: Effect of High vs Low Doses of Chloroquine Diphosphate as Adjunctive Therapy for Patients Hospitalized With Severe Acute Respiratory Syndrome Coronavirus 2 (SARS-CoV-2) Infection: A Randomized Clinical Trial
Source: JAMA Netw Open. 2020 Apr 24;3(4):e208857. doi: 10.1001/jamanetworkopen.2020.8857 (PMC12124691; doi:10.1001/jamanetworkopen.2020.8857)
Supplement: Supplement 1. — Trial Protocol [file jamanetwopen-e208857-s001.pdf]

TITLE

Protocol Title: Phase IIb study to evaluate the efficacy and safety of chloroquine diphosphate in the treatment of hospitalized patients with severe respiratory syndrome in the context of the new coronavirus (SARS-CoV2): a randomized, double-blind clinical trial.

Acronym: CloroCOVID-19

Authors: Marcus Vinícius Guimarães de Lacerda (principal researcher), Mayla Borba (co-principal researcher), Gisely Cardoso de Melo (co-investigator), Maria Paula Gomes Mourão (co-investigator), Fernando Fonseca de Almeida e Val (co-investigator), Marcia Almeida de Araújo Alexandre (co-investigator), Felipe Gomes Naveca (co-investigator), Djane Baía da Silva (collaborating researcher), Quézia de Sousa Monteiro (infectious disease specialist), Leandro Moura (infectious disease specialist), Mariana Simão Xavier (pharmacist), Marcelo Brito (pharmacist), Marcus Vinitius de Farias Guerra (collaborator), Ludhmila Abrahão Hajjar (collaborator), Jorge Souza Mendonça (collaborator), Maria Eduarda Leão de Farias (nurse), Alex Bezerra da Silva Maciel (nurse), Luiz Carlos de Lima Ferreira (pathologist)

Data and Safety Monitoring Bard (DSMB): Quique Bassat (ISGLOBAL, Barcelona, Spain), Bernardino Cláudio de Albuquerque (UFAM, Manaus, AM, Brazil), Cor Jesus (UFMT, Cuiabá, MT, Brazil) and Gustavo Romero (UnB, Brasília, DF, Brazil)

Sponsor's name and registered legal address:
Secretaria de Estado da Saúde (SUSAM)
Av. André Araújo, 701
Aleixo, Manaus – AM, 69067-375
Brazil
Telephone: (92) 3643-6300

Proponent institution:
Fundação de Medicina Tropical Dr. Heitor Vieira Dourado

Av. Pedro Teixeira, 25
Dom Pedro, Manaus - AM, 69040-000
Brazil
Telephone: (92) 2127-3555

Participating institutions:
Hospital e Pronto-Socorro Delphina Rinaldi Abdel Aziz
Av. Torquato Tapajós, 9250
Colônia Terra Nova, Manaus - AM, 69093-415
Brazil
Telephone: (92) 3090-0500

Fundação Oswaldo Cruz - Instituto Leônidas & Maria Deane
Rua Terezina, 476
Adrianópolis, Manaus - AM, 69057-070
Brazil

Telephone: (92) 3621-2323

Farmanguinhos (Fiocruz-RJ)
Av. Brasil, 4365
Manguinhos, Rio de Janeiro - RJ, 21040-900
Brazil
Telephone: (21) 2598-4242

Fundação de Vigilância em Saúde (FVS) - Laboratório Central de Saúde Pública (LACEN)
R. Emílio Moreira, 528
Centro, Manaus - AM, 69020-040
Brazil
Telephone: (92) 3182-8750

Hospital Santa Júlia
Av. Alvaro Maia, 510
Centro, Manaus - AM, 69025-050
Brazil
Telephone: (92) 2121-9000

Fundação Municipal de Saúde de Niterói
Researchers: André Siqueira
Rua Visconde de Sepetiba, 987, 8° andar
Centro, Niterói – RJ, 24020-220
Brazil
Telephone: (21) 2716-5800

Hospital Universitário Maria Aparecida Pedrossian
Researchers: Julio Henrique Rosa Croda e Mariana Croda
Av. Sen. Filinto Müler, 355
Vila Ipiranga, Campo Grande - MS, 79080-190
Brazil
Telephone: (67) 3345-3000

Hospital Regional de Mato Grosso do Sul
Researchers: Julio Henrique Rosa Croda e Mariana Croda
Av. Eng. Lutero Lopes, 36
Conj. Aero Rancho, Campo Grande - MS, 79084-180
Brazil
Telephone: (67) 3378-2500

Summary

1. PROTOCOL SUMMARY 6
SYNOPSIS 6
1.1 Justification for the proposed clinical study .... Error! Bookmark not defined.
1.2 Study design 6
1.3 Sample calculation 7
1.4 Study objectives .... Error! Bookmark not defined.
1.4.1 Primary objective 7
1.4.2 Secondary objectives 7
1.5 Study outcomes .... Error! Bookmark not defined.
1.4.1 Primary outcome 8
1.4.2 Secondary outcomes 8
1.6 Inclusion criteria .... Error! Bookmark not defined.
1.6 Exclusion criteria 9
1.7 Study phase 9
1.8 Study population 9
1.9 Study location 9
1.10 Study intervention 10
1.11 Study duration 11
1.11.1 Follow-up duration 11
1.11.2 Safety 11
1 .13 Study flowchart 16
2. INTRODUCTION 17
2.1 Rationale of the study 17
2.2 Background 17
2.3 Risk/benefit assessment 20
2.3.1 Potential known risks 20
2.3.1.1 Toxic manifestations 21
Toxic manifestations related to the use of chloroquine are listed below: 21
2.3.1.2 Risks involving blood collection from biological samples 21
2.3.2 Unknown risks 22

2.3.3 Risks to privacy 22
2.3.4 Potential benefits 22
3. OBJECTIVES AND OUTCOMES 22
4. STUDY DESIGN 24
4.1 General design of project 24
4.2 Scientific basis for the study design 24
4.3 Placebo justification 25
5. STUDY POPULATION 25
5.1 Sample calculation 25
5.2 Inclusion criteria .... Error! Bookmark not defined.
5.3 Exclusion of specific populations 26
5.4 Inclusion of vulnerable participants 26
5.5 Recruitment and retention strategies 26
5.5.1 Recruitment 27
5.5.2 Retention 27
5.5.3 Compensation plan for subjects 27
5.5.4 Costs 27
6. STUDY DRUG 28
6.1 Description of the study drug .... Error! Bookmark not defined.
6.2 Dose and administration 29
6.3 Dose escalation 29
6.4 Dose modifications and concomitant drugs 30
6.5 Acquisition 30
6.6 Preparation, handling, storage and responsibilities 30
6.7 Collection and/or destruction of medicines 31
6.8 Accounting and adherence to treatment 31
7. MEASURES TO MINIMIZE BIAS: RANDOMIZATION AND BLINDNESS ...... Error! Bookmark not defined.
7.1 Compliance with the study intervention 32
7.2 Concomitant therapy 32
7.3 Salvage drug 32
8. DISCONTINUATION OF THE INTERVENTION AND WITHDRAWAL FROM THE STUDY 32
8.1 Criteria for stopping and discontinuing study intervention 32

8.1.1 Administration interruption 33
8.1.2 Interruption of study for safety reasons 33
8.1.3 Withdrawal from randomized treatment or from study 33
8.1.4 Study drug discontinuation 33
8.1.5 Withdrawal of patients from the study 34
8.1.6 Study interruption 34
9. STUDY EVALUATIONS AND PROCEDURES 35
9.1 Definition of source documents 35
9.2 Security instructions ..... Error! Bookmark not defined.
9.5 Monitoring of adverse events 35
9.6 Adverse Event (AE) and Serious Adverse Event (SAE) definitions 36
9.7 Post-mortem exams ..... Error! Bookmark not defined.
10. STATISTICS 36
10.1 Analytical plan 36
10.2 Sample calculation and interim analysis 37
11. SUPPORTING DOCUMENTATION AND OPERATIONAL CONSIDERATIONS 38
11.1 Regulatory, ethical and supervisory considerations of the study 38
11.2 Informed consent process 39
11.3 End and shutdown of study 40
11.4 Confidentiality and privacy 40
11.5 Secondary use of samples and stored data 41
11.6 Clinical monitoring 41
11.7 Data management and record keeping 42
11.8 Protocol deviations 43
11.9 Data publishing and sharing policy 43
11.10 Conflict of interest policy 44
11.11 Amendments to the clinical study protocol 44

1. PROTOCOL SUMMARY

SYNOPSIS

1.1 Justification for the proposed clinical study

In December, 2019, the Wuhan Municipal Health Committee in China identified an outbreak of viral pneumonia cases of unknown cause. Coronavirus RNA was quickly identified in some of these patients. This new coronavirus was called **SARS-CoV2** and the disease caused by that virus, **COVID-19**. To date, there are no approved therapeutic agents available for the specific treatment of coronavirus. In the absence of a known effective therapy in a global public health emergency, there is little evidence about the potential antiviral effect of chloroquine (CQ) in most coronaviruses, including SARS-CoV-1. We intend to investigate the efficacy and safety of treatment with CQ diphosphate in hospitalized patients with severe acute respiratory syndrome (SARS) within the scope of SARS-CoV2. Preliminary trials of CQ repositioning in the treatment of COVID-19 have been encouraged. As of the date of this version, there were 23 clinical trials registered with this theme, in progress in China (research conducted on *clinicaltrials.gov* and *Chictr*). However, with the reduction in the number of cases in the country, the chance of conclusions that may be used for treatment in other continents is low.

1.2 Study design

This is a randomized, double-blind, placebo-controlled clinical trial. A total of 440 individuals aged between 18 and 80 years, diagnosed with severe respiratory disease, hospitalized in one of the hospitals participating in the study, with suspected SARS-CoV2, will be randomized into two treatment groups at a 1:1 ratio, with group 1 receiving CQ 600mg twice a day for 10 days and group 2 receiving CQ 450mg twice a day on the first day and 450mg once a day for the next 4 days. In order to blind study team and participants, placebo tablets will be used to standardize group 2 treatment for 10 days as group 1. According to the Contingency Plan for coping with Covid-19 in Amazonas, the Hospital e Pronto Socorro Delphina Rinaldi Abdel Aziz Hospital (HPSDRAA), in the north of Manaus, is the reference unit for the admission of serious cases of the new virus. The unit currently has 50 ICU beds, which can increase to a

capacity of 150 ICU beds, 335 beds in total. The hospital also has trained multiprofessional human resources and adequate infrastructure to serve this population. It is the largest hospital in the country dedicated exclusively to the admission of patients with COVID-19, and is publically-privately managed.

1.3 Sample calculation

The sample for the primary outcome (reduction in lethality) was calculated assuming a 20% lethality incidence in critically ill patients not using CQ (historical control) and that both arms of CQ would be equally able to reduce lethality by at least 50%. Thus, considering a test of differences in proportions between 2 groups of the same size, 80% power and 5% alpha, 394 participants were needed (197 per group). Adding 10% of losses, the final sample of 440 participants was obtained. Sample calculation was performed in the R statistical package (v3.6.1), with the functions implemented in the TrialSize and gsDesign packages.

1.4 Study objectives

1.4.1 Primary objective

Assess whether the therapeutic regimen of group 1 reduces mortality by 50% in the study population compared to group 2.

1.4.2 Secondary objectives

1. Assess mortality on days 7, 14 and 28 after hospitalization;

2. Evaluate indicators of clinical worsening (use of supplemental oxygen, ICU stay, length of stay in the ICU, length of hospital stay, need for intubation and time on mechanical ventilation, radiological alterations and time to improve the radiological and resonance pattern);

3. Assess the percentage of SARS-CoV2 detection in oropharyngeal swab, nasal and blood swab samples on days 1, 3, 5, 8, 11, 14 and 28, in the study population;

4. Quantify the virus that causes SARS-CoV2 in oropharyngeal swab, nasal and blood swab samples on days 1, 3, 5, 8, 11, 14 and 28, in the study population;

5. Cumulative incidence of adverse events (AEs);

6. Cumulative incidence of grade 3 and 4 serious adverse events (SAEs);

7. Discontinuation or temporary suspension of treatment (for any reason);

8. Alterations in white cell count, hemoglobin, platelets, creatinine, glucose, total bilirubin, ALT and AST over time;

9. In case of mortality, describe organic alterations via autopsy.

1.5 Study outcomes

1.4.1 Primary outcome

- 50% mortality reduction by day 28.

1.4.2 Secondary outcomes

1. Mortality on days 7 and 14;
2. Clinical status of the study subject on days 14 and 28;
3. Daily clinical status during hospitalization;
4. Time on supplemental oxygen (if applicable);
5. Time on mechanical ventilation (if applicable);
6. Length of hospitalization;
7. Data and cause of death (if applicable);
8. Incidence of adverse events;

9. Incidence of serious adverse events (grades 3 and 4).

1.6 Inclusion criteria

1. Adult 18 years of age or older, at the time of inclusion (**children under 18 will not be included due to the recognized lower lethality in previous published studies, and the difficulty of consent in the context of a public health emergency**);
2. Hospitalized;
3. And presents with:

- Respiratory rate > 24 bpm AND/OR
- Heart rate > 125 bpm (in the absence of fever) AND/OR
- Peripheral oxygen saturation <90% in ambient air AND/OR
- Shock (defined as MAP <65 mmHg, requiring a vasopressor or oliguria or lowering the level of consciousness)

1.6 Exclusion criteria

(Does not apply).

1.7 Study phase

Phase IIb.

1.8 Study population

Groups 1 and 2: Hospitalized adult patients (≥18 years old) with severe respiratory syndrome.

1.9 Study location

- Delphina Rinaldi Abdel Aziz Hospital and A & E (HPSDRAA), Manaus, AM, Brazil
- Hospital Santa Júlia, Manaus, AM, Brazil
- Fundação Municipal de Saúde, Niterói-RJ, Brazil

- Hospital Universitário Maria Aparecida Pedrossian, Campo Grande-MS, Brazil
- Hospital Regional de Mato Grosso do Sul, Campo Grande-MS, Brazil

1.10 Study intervention

**Group 1 (higher dosage – 10 days)**		
	**1st daily dose**	**2a daily dose**
**D1 – D10**	**4 tablets of 150mg (600mg)**	**4 tablets of 150mg (600mg)**
**Grupo 2 (lower dosage – 5 days CQ + 5 days placebo)**		
**D1 (Loading dose)**	**3 tablets of 150mg (450mg) + 1 placebo tablet**	**3 tablets of 150mg (450mg) + 1 placebo tablet**
**D2 – D5**	**3 tablet of 150mg + 1 placebo tablets**	**4 placebo tablets**
**D6 – D10**	**4 placebo tablets**	**4 placebo tablets**

*both chloroquine and placebo are produced by Farmanguinhos. Both have the same physical presentation (dimensions and color).

We emphasize that there is proven evidence regarding chloroquine for the study and that to date it is not recommended in the official protocol. The research team will collect care data and will not perform patient management per se. The management of the participants will be carried out by the hospital's medical team and is dynamic, being changed according to new evidence and recommendations from the Ministry of Health.

Treatment *per os* (or nasogastric tube in case of orotracheal intubation - OTI) for 10 days, if necessary (patient on mechanical ventilation), regardless of the allocation group.

In Group 1, as the dose chosen is the highest used in the China experiment, there will be no attack dose on day 1. The choice of chloroquine diphosphate is due to its routine use for malaria by *Plasmodium vivax* (however at a lower dose and for only three days), which already has regular distribution logistics, even to remote areas and is produced nationally by a state laboratory (Farmanguinhos), and is not available for purchase commercial pharmacies. Hydroxychloroquine is used for the treatment of autoimmune diseases such as systemic lupus erythematosus, rheumatoid arthritis, undifferentiated disease, mixed disease and some

cutaneous vasculitis. It is less toxic in the context of chronic use, but for short periods of treatment, both medications are very safe, even in pregnant women.

1.11 Study duration

The study will last for the period of appearance of cases (expected between April 6th and August 31st, 2020), or until the sample is completed, or until the interim analysis shows a clear benefit or harm. The period was calculated based on our modeling of the number of cases in Manaus, as shown below.

To obtain a possible scenario of infected by COVID-19 in the city of Manaus, we used simulation using the SIR model (Susceptible, Infected and Recovered) to have an idea of the probable period when we will have the peak of infection in the city of Manaus. The parameters used for the modeling were as follows: R0 = 2.5, from studies in China; the Susceptible rate 0.92, the infected rate 0.01 and the Recovered rate 1/14. The first case found in Manaus was diagnosed on March 11th, 2020.

The obtained result can be seen in the graph below.

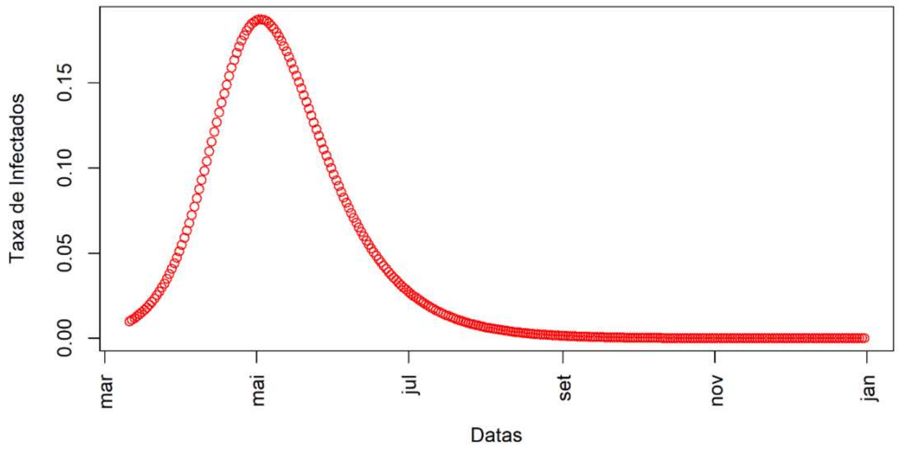

1.11.1 Follow-up duration

An individual will complete the study in 28 days, starting from their inclusion.

1.11.2 Safety

Given the severity of the disease COVID-19, there is no study pre-specifying criteria for interrupting follow-up. The protocol team will review blind AE/SAE data every 2 weeks. If there is a worrying number of unexpected AEs, the researcher will request a review of the non-hidden security data in an ad hoc meeting with the external security monitoring committee, which should take place in real time, via videoconference.

1.12 Table 1: Schedule for evaluation throughout the study

**Description of the procedures according to the protocol**[μ]	D0*	D1[∞]	D2	D3	D4	D5	D6	D7	D8	D9	D10	D11	Dx	D14[#]	D28[#]
Application of ICF[τ]	x														
Review of eligibility criteria[a]	x														
Randomization[b]	x														
Demography, medical history, including comorbidities (HAS, DM, dG6PD) and co-infections (HIV, TB, hepatitis)[c]	x														
Physical exam[d]	x														
Clinical evaluation[e]	x	x	x	x	x	x	x	x	x	x	x	x	x	x	x
Evaluation of concomitant medication[f]	x	x	x	x	x	x	x	x	x	x	x	x	x	x	x
12-lead ECG[g]	x	x	x	x	x	x	x	x	x	x	x	x	x	x	x
Blood sample collection for hematology and biochemistry[h]	x	(x)	(x)	(x)	(x)	(x)	(x)	(x)	(x)	(x)	(x)	(x)	(x)	(x)	(x)
Blood sample collection for virology and storage[i]	x	x		x		x		x				x	x	x	x
Oropharyngeal Swab and Nasal Swab	x	x		x		x		x		x		x	x	x	x
Record of adverse events (serious and non-serious)[j]	x	x	x	x	x	x	x	x	x	x	x	x	x	x	x
Assessment of the primary outcome[k]								x						x	x
Record of secondary outcomes[l]		x	x	x	x	x	x	x	x	x	x	x	x	x	x

Respiratory evaluation[m]	(x)	(x)	(x)	(x)	(x)	(x)	(x)	(x)	(x)	(x)	(x)	(x)	(x)	(x)	(x)
Radiological evaluation[n]	(x)	(x)	(x)	(x)	(x)	(x)	(x)	(x)	(x)	(x)	(x)	(x)	(x)	(x)	(x)

μ – All data from the study procedures will be collected and entered into the RedCap system;
* Screening and Randomization may occur on the same day. All procedures must be performed before the study intervention;
∞ – Evaluations and collections should take place after 24 hours of administration of the investigational product;
τ – The Informed Consent Form will be obtained by the study nurse directly from the participant, or in case of no possibility of this occurring (example: loss of level of consciousness), from a family member or legal guardian.
Dx – Days of hospitalization after completion of treatment;
# – Participant may be hospitalized or discharged. In the latter case, there will be no outpatient return visit and the main outcome will be assessed by telephone. Note: all procedures will be performed during the hospitalization period and will be interrupted if the participant is discharged;
a – Inclusion/exclusion criteria will be fully assessed at visit D1, prior to inclusion. The diagnosis of SARS-CoV2 will occur *a posteriori*.
b – Randomization will take place in blocks of 4 with 110 participants each. There will be interim reviews when 25%, 50% and 75% of participants are included;
c – A complete medical history will be obtained prior to randomization to include assessments of cardiovascular, metabolic (for example, Type I or II diabetes mellitus), psychiatric (such as depression), renal (such as nephrolithiasis, nephropathy, renal failure) and bone disorders;
d – Physical examination will consist of evaluation of vital signs (heart rate, respiratory rate, systolic, diastolic and mean blood pressure, peripheral oxygen saturation) and signs and symptoms present;
e – Assessments to highlight any visual and/or mental alterations resulting from chloroquine;
f – Avoid administration of other drugs known to prolong the QT interval (ie quinolones, macrolides, ondansetron), as well as various antiarrhythmic, antidepressant and antipsychotic drugs; Avoid administration of other drugs known to prolong the QT interval (ie quinolones, macrolides, ondansetron), as well as various antiarrhythmic, antidepressant and antipsychotic drugs;
g – A 12-lead ECG will be performed in a semi-supine position after resting for at least 5 minutes. The intention is to exclude the development of prolongation in the QT interval or bradycardia;
h – Blood samples will be collected to assess the COVID-19 infection and perform laboratory tests requested by the doctor. In the case of Manaus, the evaluation of the infection by COVID-19 will take place at LACEN-FVS and the others will take place at the Delphina Rinaldi Abdel Aziz Hospital and A & E. In Niterói-RJ all tests will be performed at the hospital. In Mato Grosso do Sul, the evaluation of infection by COVID-19 will take place at LACEN-MS and any others will take place at participating hospitals;

i – Blood samples will be collected for storage in a biorepository for further evaluation of the immune response profile;
j – Evaluated according to the DAIDS table for clinical and laboratory events;
k – Evaluated and recorded in the hospital's electronic medical record and inserted in the RedCAP system;
l – Evaluated and recorded in the hospital's electronic medical record and inserted in the RedCAP system;
m – If a patient needs it, orotracheal intubation will be performed and the parameters of the mechanical ventilator will be recorded daily;
n – Radiology, tomography or nuclear magnetic resonance exams will be performed according to medical request and New Coronavirus Treatment Protocol (2019-nCOV)
( ) – denotes optional procedure, to be evaluated by the health professional according to clinical needs

1 .13 Study flowchart

The therapeutic schematic and mortality assessments are shown in figure 1.

Figure 1. Therapeutic scheme for monitoring patients using chloroquine in lower and higher doses.

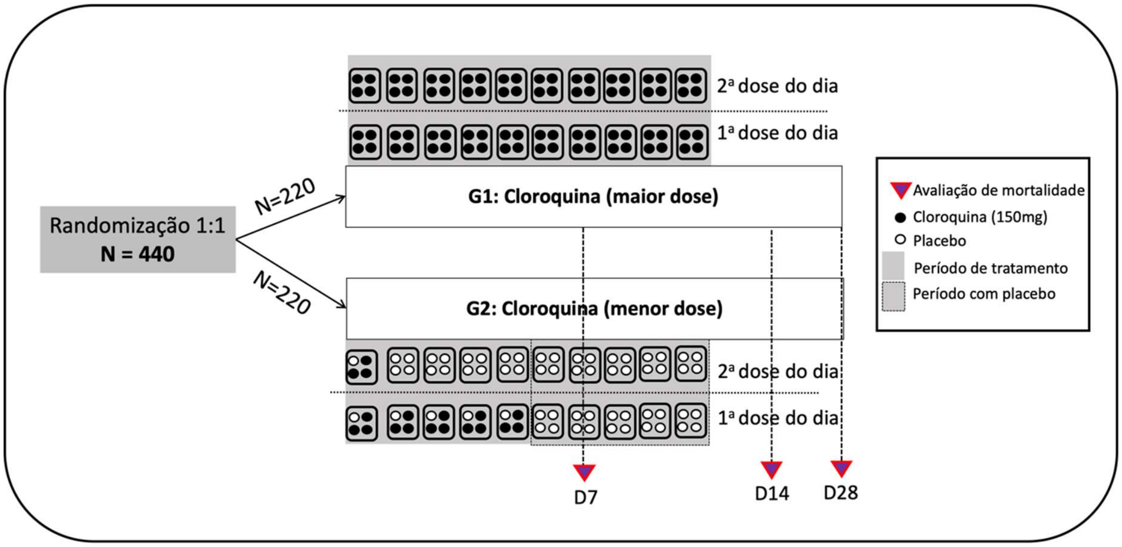

Dosage:

Group 1 (higher dosage – 10 days)		
	**1st daily dose**	**2ª daily dose**
**D1 – D10**	**4 tablets of 150mg (600mg)**	**4 tablets of 150mg (600mg)**
**Group 2 (lower dosage – 5 days CQ + 5 days placebo)**		
**D1 (Loading dose)**	**3 tablets of 150mg (450mg) + 1 tablets of placebo**	**3 tablets of 150mg (450mg) + 1 tablets of placebo**
**D2 – D5**	**3 tablets of 150mg + 1 tablets of placebo**	**4 tablets of placebo**
**D6 – D10**	**4 tablets of placebo**	**4 tablets of placebo**

Administration: Oral (or via nasogastric tube in case of orotracheal intubation - OTI)

Placebo: pills without active ingredient but with the same appearance (there will be no exclusive placebo arm) will be used to standardize treatment.

2. INTRODUCTION

2.1 Rationale of the study

COVID-19 is a respiratory disease caused by a new coronavirus (SARS-CoV2) and causes substantial morbidity and mortality. There is currently no vaccine to prevent COVID-19 or infection by SARS-CoV-2 or a therapeutic agent to treat COVID-19. This clinical trial was designed to evaluate the therapeutic effect of chloroquine for the treatment of hospitalized adult patients with severe respiratory syndrome.

2.2 Background

Coronavirus is one of the main pathogens that targets the human respiratory system. Previous outbreaks of coronavirus (CoVs) include severe acute respiratory syndrome (SARS) -CoV and Middle East respiratory syndrome (MERS)-CoV, and are characterized as agents that pose a threat to public health[1]. The first cases of the new coronavirus 2019 (COVID-19) were reported in December, 2019, when a group of patients was admitted to hospitals with an initial diagnosis of pneumonia of unknown etiology[2]. From December 18th to 29th, 2019, five patients were hospitalized with acute respiratory distress syndrome and one of them died[3]. On January 2nd, 2020, 41 hospitalized patients were identified as having confirmed COVID-19 infection, less than half of these patients had underlying diseases, including diabetes, hypertension and cardiovascular disease[3]. As of January 22nd, 2020, a total of 571 cases were reported in 25 provinces in China[4]. On January 25th, 2020, a total of 1975 cases were confirmed in mainland China, with a total of 56 deaths[5]. On January 30, 773 cases were confirmed in China and 90 other cases were reported in several countries including Taiwan, Thailand, Vietnam, Malaysia, Nepal, Sri Lanka, Cambodia, Japan, Singapore, the Republic of Korea, United Arab Emirates, the United States, the Philippines, India, Australia, Canada, Finland, France and Germany. The case mortality rate was estimated at 2.2% (170/7824)[6].

The first confirmed case of COVID-19 infection in the United States, on January 19th, led to the description, identification, diagnosis, clinical course and treatment of this case, which includes the patient's initial mild symptoms on presentation and progression to pneumonia on the day 9 of the disease[7]. Italy had 12,462 confirmed cases according to the Istituto Superiore

di Sanità on March 11th and 827 deaths. The average age of those who died in Italy was 81 and more than two-thirds of those patients had diabetes, cardiovascular disease or cancer, or were ex-smokers[8].

As of March 15th, the World Health Organization (WHO) registered 51,174 confirmed cases, including 15,384 serious cases and 1,666 cases of death in China. Globally, the number of confirmed cases at the time of writing was 51,857 in 25 countries (https://www.who.int/docs/default-source/coronaviruse/situation-reports). In Brazil, there are 428 confirmed cases in 20 states and the DF. The most affected states are São Paulo, with 240 confirmed cases (56.1%), followed by RJ with 45 (10.5%) and the Federal District with 26 cases (6.1%). There are already seven dead in Brazil, five in SP and two in RJ (March 19th, 2020) (http://platform.saude.gov.br/novocoronavirus/). The cases have increased and there is currently no specific antiviral therapy for coronavirus infections. Few treatment studies have been carried out because most strains of human coronavirus cause self-limiting diseases and care is in the form of support. After the severe acute respiratory syndrome (SARS), the coronavirus was identified in 2002 and caused a major global outbreak, there was a growing interest in the development of specific therapeutic agents. Thus, since the SARS outbreak, new therapeutic agents targeting viral entry, proteins, proteases, polymerases and methyltransferases have been tested. However, none of them have been shown to be effective in clinical trials. Recent publications have drawn attention to the possible benefit of chloroquine, a widely used antimalarial drug, in the treatment of patients infected with SARS-CoV2[9–15]

Chloroquine sulfate and phosphate salts (chloroquine diphosphate-CQ) are used as antimalarial drugs. Hydroxychloroquine (HCQ), a derivative of CQ, was first synthesized in 1946 by the introduction of a hydroxyl group in CQ and proved to be less toxic (~ 40%) in its prolonged use, and is therefore recommended to treat chronic diseases, such as systemic lupus erythematosus and rheumatoid arthritis[16]. Both chloroquine diphosphate (CQ) and hydroxychloroquine (HCQ) have a bitter taste, but are generally very well tolerated. **CQ can accumulate in tissues, especially the eye, causing retinal toxicity, associated with the use of high doses and prolonged use**[17,18]. Myopathy has also been associated with the use of CQ[19]. For the treatment of malaria, CQ is recommended, in particular, due to its low cost; moreover, for the treatment of the disease few doses are used, and in safe concentrations[19]. In In Brazil,

the Ministry of Health guidelines indicate chloroquine diphosphate and other antimalarial drugs as the treatment for malaria and makes them available throughout the country in the health care centers of the Unified Health System (SUS). CQ diphosphate is produced in Brazil and is part of strategic pharmaceutical assistance programs. It is distributed in all states through a well-established distribution network for the treatment of vivax malaria (cause of 90% of malaria in Brazil).

The *in vitro* antiviral activity of CQ was identified in the late 1960s[20],[21] and the growth of many different viruses can be inhibited in cell culture by both chloroquine and hydroxychloroquine, including SARS-CoV2[15]. Some evidence of activity in mice has been found for a variety of viruses, including the human coronavirus OC43, the enterovirus EV-A71 and the Zika virus and influenza A H5N1[22–25]. Wang et al. showed the effect of chloroquine in vitro, using SARS-CoV2 infected Vero E6 cells at a multiplicity of infection (MOI) of 0.05. The study demonstrated that CQ was highly effective in reducing viral replication, with an effective concentration (EC) 90 of 6.90 µM that can be easily achieved with standard dosing, due to its favorable penetration into tissues, including that of the lung[26]. The authors described that CQ is known to block infection by the virus, increasing the endosomial pH and interfering with the glycosylation of the SARS-CoV cell receptor. The authors also speculated on the possibility that the known immunomodulating effect of the drug may increase the antiviral effect *in vivo*[26]. Thus, according to these authors, CQ is highly effective in controlling infection by 2019-nCoV *in vitro* and the safety history of CQ suggests that it should be evaluated in human patients suffering from the new coronavirus disease. In a recent publication, Gao et al.[27] demonstrated, based on the use of chloroquine by 100 patients, that the effect of CQ diphosphate is superior to the control treatment in inhibiting the exacerbation of pneumonia, improving pulmonary imaging findings and promoting a negative conversion of the virus and reducing the course of the disease.

The *Multicenter Collaboration Group of Department of Science and Technology of Guangdong Province* and the *Health Commission of Guangdong Province*[28] based on *in vitro* evidence and data from unpublished clinical experiments recommended CQ diphosphate tablets, at a dose of 500 mg twice daily for 10 days, for patients with mild, moderate and severe cases of SARS-CoV2 pneumonia, as long as there were no contraindications for the medication. However, to ensure the safe use of the drug, due to its toxic effects, the use of

several precautions was recommended, including blood tests to exclude the development of anemia, thrombocytopenia or leukopenia, as well as serum electrolyte disturbances and/or liver and kidney dysfunction. They recommended (i) a routine electrocardiogram, in order to exclude the development of prolongation in the QTc interval or bradycardia, (ii) conducting interviews to highlight potential visual and/or mental problems and (iii) avoiding administration of other drugs known to prolong the QTc interval (i.e. quinolones, macrolides, ondansetron), as well as various antiarrhythmic, antidepressant and antipsychotic medications.

The CDC[29] has a public document on its website suggesting to treat serious infections through hospital admission with oxygen therapy and CQ. The suggested CQ regimen for adults consists of 600 mg of CQ base (6 tablets A-CQ 100 mg), followed by 300 mg after 12 h on day 1, then 300 mg × 2 / given orally on days 2 to 5 days. This document states the need to stop treatment on day 5 to reduce the risk of side effects, considering the drug's long half-life (30 h). Another guidance document from the Italian Society of Infectious Diseases and Tropical Disease (Lombardy section) recommends using CQ 500 mg × 2 a day or HCQ 200 mg for 10 days, although treatment can vary from 5 to 20 days, according to clinical severity.

The fact that so many studies are conducted in parallel using CQ and HCQ suggests that the scientific community is making a great effort to clarify the role of these drugs in reducing the mortality associated with COVID-19, but this effort is probably not yet sufficiently well-coordinated. Thus, clinical trials that study potential pharmacological treatments for COVID-19 must be performed, so that the safety and efficacy of CQ is demonstrated. Related scientific publications are important and timely in view of the growing number of infections and the absence of specific, licensed drugs.

2.3 Risk/benefit assessment

2.3.1 Known potential risks

2.3.1.1 Toxic manifestations

Toxic manifestations related to the use of chloroquine are listed below:

1. Cardiovascular alterations (hypotension, vasodilation, suppression of myocardial function, cardiac arrhythmias, cardiac arrest); rare electrocardiogram alterations, such as inversion of the T wave and enlargement of the QRS complex, AV block, cardiomyopathy;

2. Alterations of the central nervous system, the most common being: visual accommodation disorder, blurred vision, headache, fatigue, nervousness, anxiety, apathy. Rare alterations include irritability, agitation, aggression, confusion, personality change, depression and psychic stimulation, peripheral neuritis, neuromyopathy;
3. Digestive system, the most common being: gastrointestinal irritation, nausea, vomiting, stomatitis and rare fulminant liver failure;
4. Hematological: rare alterations include neutropenia, agranulocytosis, aplastic anemia, thrombocytopenia;
5. Renal, rare alterations include renal failure in patients with G6PD deficiency;
6. Dermatological: most common alterations include itching, dark blue coloration of the mouth, skin and nails, hair whitening, hair loss, skin rash.
7. Other common alterations include corneal opacity, acute attack of porphyria and psoriasis in susceptible people. Rare alterations include ototoxicity, muscle weakness. High daily doses (> 250 mg), resulting in cumulative doses of more than 1 g/kg of base chloroquine, can result in irreversible retinopathy and ototoxicity.

It is noteworthy that the use of the drug will be for a short time and, as such, it has rarely been associated with the risks described above, which are only ever seen in prolonged use of the medication.

2.3.1.2 Risks involving blood collection from biological samples

Blood collection may cause some discomfort in some people, dizziness, bleeding, bruising or swelling at the collection site. It can rarely cause fainting or infection.

2.3.2 Unknown risks

Other side effects not yet known at this time may happen during the study. All medications have a possible risk of an allergic reaction, which will be treated properly if or when it occurs.

2.3.3 Risks to privacy

Individuals will be asked to provide personal health information. All attempts will be made to keep this information confidential within the limits of the law. However, there is a possibility of loss of confidentiality. All study records will be kept in a closed file or in a locked room. Electronic files will be password protected. Only persons involved in conduct, supervision, monitoring or auditing will be allowed access to the information collected. No publication in this study will use information that could identify subjects by name.

2.3.4 Potential benefits

Information from this study can help treatment and management of hospitalized patients and will help future patients with COVID-19, reducing mortality caused by COVID-19 in hospitalized patients.

3. OBJECTIVES AND OUTCOMES

The general objective of the study is to evaluate the clinical efficacy, safety of therapy and reduction of mortality in hospitalized adult patients with severe respiratory syndrome using two different dosages of chloroquine. Objectives and outcomes are shown in table 2.

Table 2: Objectives and outcomes.

**Objectives**	**Outcomes**
**Primary**	
To assess whether the therapeutic regimen of Group 1 reduces mortality by 50% in the study population compared to Group 2.	Reduction of mortality by 50%
**Secondary**	
- Assess mortality on days 7, 14 and 28 after hospitalization;<br>- Assess clinical improvement (use of supplemental oxygen, ICU stay, length of stay in the ICU, length of hospital stay, need for OTI and time on mechanical ventilation, radiological alterations and time taken to improve the radiological and resonance pattern);<br>- Assess the percentage of SARS-CoV2 detection in OPS and blood samples on days 1, 3, 5, 7, 14 and 28 in the study population;<br>Quantify the SARS-CoV2 virus in oropharyngeal swab, nasal swab, OPS and blood samples on days 1, 3, 5, 7, 14 and 28 in the study population;<br>- Alterations in white cell count, hemoglobin, platelets, creatinine, glucose, total bilirubin, ALT and AST over time.<br>- Cumulative incidence of serious adverse events (SAEs);<br>- Cumulative incidence of grade 3 and 4 adverse events (AEs);<br>- Discontinuation or temporary suspension of treatment (for any reason);	- Mortality on days 7 and 14<br>- Clinical status of the subject on days 14 and 28<br>-Daily clinical status during hospitalization<br>- Need for and duration of supplementary oxygen<br>- Need for and duration of mechanical ventilation<br>- Length of hospitalization<br>- Data and cause of death (if applicable)<br>- Prevalence of grade 3 and 4 adverse events;<br>- Prevalence of serious adverse events;<br>- Leukogram, hemoglobin, platelets, creatinine, CK, glucose, total bilirubin, ALT and AST on days 1, 3, 5, 7 (while hospitalized); and on days 14 and 28 (if patient is able to return to the clinic or is still hospitalized);<br>- Negative presence of the virus in respiratory secretion;<br>- Viremia by RT-PCR;<br>- Verification of anatomical-pathological alterations in cases that evolved to death.

- In case of mortality, describe organic alterations based on a complete or minimally invasive autopsy.	

4. STUDY DESIGN

4.1 General design of the project

This is a double-blind, randomized, placebo-controlled clinical trial. A total of 440 individuals, aged 18 years or older, diagnosed with severe respiratory disease, without contraindications to CQ, hospitalized in the HPSDRAA, with suspected SARS-CoV2, will be randomized into two treatment groups, in the ratio of 1:1, with group 1 receiving CQ 600mg twice a day for 10 days or group 2 receiving CQ 450mg twice a day on the first day and 450mg once a day the following 4 days. In order to blind study team and participants, placebo tablets will be used to standardize group 2 treatment for 10 days as group 1. Both groups will receive drugs via oral administration or nasogastric tube in case of orotracheal intubation. Chloroquine and placebo will be donated by Farmanguinhos on an emergency basis. Clinical and laboratory data during hospitalization will be used to assess the efficacy and safety outcomes. Blood samples and oropharyngeal swabs (OPS) will be obtained on days 1, 3, 5, 7, 14 and 28 (while hospitalized).

4.2 Scientific basis for the study design

Currently, there is no specific antiviral therapy for coronavirus infections. Few treatment studies have been conducted because most strains of human coronavirus cause self-limiting diseases and care is of a supportive nature. After the severe acute respiratory syndrome (SARS), the coronavirus was identified in 2002 and caused a major global outbreak, there has been a growing interest in the development of specific therapeutic agents. Since the SARS outbreak, new therapeutic agents which target the entry of viral proteins, proteases, polymerases and methyltransferases have been tested, however, none of them have been shown

to be effective in clinical trials. Thus, in the absence of a known effective therapy, the situation of a global public health emergency and evidence regarding the potential chloroquine inhibitor (CQ) in most coronaviruses, including SARS-CoV1 and others of a clinical nature, we intend to investigate the efficacy and the safety of CQ diphosphate in the treatment of hospitalized patients with severe respiratory syndrome due to SARS-CoV2. Preliminary trials of CQ repositioning in the treatment of COVID-19 have been encouraging.

This study uses an adaptable design that assesses a safe and effective therapeutic agent (chloroquine) for COVID-19 during the pandemic. The research product (chloroquine diphosphate) is easy to obtain, since it is produced on a large scale within the national territory and is widely distributed to all states by the National Malaria Program, of the Brazilian Ministry of Health. In addition, as the cases have increased, the low cost of chloroquine diphosphate and the free distribution by SUS is a great benefit. Randomization and blinding are essential to establish the effectiveness of chloroquine. In addition, by collecting clinical and virological data from enrolled patients using a standardized timeline and appropriate data collection instruments, we will also be able to provide valuable information about the clinical course and morbidities associated with severe COVID-19 in a diverse group of adult patients.

4.3 Placebo justification

The placebo will be used in this study as a mechanism for blinding participants, doctors and other professionals in the study.

5. STUDY POPULATION

5.1 Sample calculation

The sample for the primary outcome (reduction in lethality in patients with severe respiratory virus treated with a high dosage of chloroquine compared to a low dosage) was calculated based on the following assumptions:

1. The 20% lethality incidence in critically ill patients per year in the low dosage group;

2. High dosage chloroquine will be able to reduce lethality by at least 50% compared to low dosage chloroquine;

Thus, considering a test of differences in proportions between 2 groups of the same size, with power of 80% and alpha of 5%, 394 participants will be needed (197 per group). Considering 10% losses, the sample size is 440 participants.

5.2 Inclusion criteria

1. Adult aged 18 years or older, at the time of inclusion **(children under 18 will not be included due to the recognized lower lethality in previous published studies, and the difficulty of consent in the context of a public health emergency)**;
2. Hospitalized;
3. And presents with clinical suspicion of COVID-19 AND:

- Respiratory rate > 24 bpm AND/OR
- Heart rate > 125 bpm (in the absence of fever) AND/OR
- Peripheral oxygen saturation <90% in ambient air AND/OR
- Shock (defined as MAP <65 mmHg, requiring a vasopressor or oliguria or lowering the level of consciousness)

5.3 Exclusion of specific populations

Children and adolescents will be excluded.

5.4 Inclusion of vulnerable participants

N/A.

5.5 Recruitment and retention strategies

5.5.1 Recruitment

It is expected that patients with COVID-19 will come to the HPSDRAA, in the northern zone of Manaus, a referral hospital for hospitalization of serious cases of the new virus, as provided for in the contingency plan to confront Covid-19 in Amazonas. Therefore, no further efforts are needed to recruit potential subjects. Recruitment efforts may, however, include the dissemination of information about this study to medical professionals and health staff at the hospital.

Patients with respiratory syndrome will be assessed for eligibility. The screening will begin with a brief discussion with the study team. Information about the study will be presented to potential subjects (or legally authorized representatives) and questions will be asked to determine possible eligibility.

As an additional recruitment strategy, centers included will be:

- Hospital Santa Júlia, Manaus-AM
- Fundação Municipal de Saúde, Niterói-RJ
- Hospital Universitário Maria Aparecida Pedrossian, Campo Grande-MS
- Hospital Regional de Mato Grosso do Sul, Campo Grande-MS

5.5.2 Retention

Not applicable, as all follow-up takes place during the patient's hospitalization period and the outcomes are discharge or death.

5.5.3 Compensation plan for subjects

There will be no financial compensation. All assistance will be provided free of charge by the institutions, part of the Unified Health System (SUS).

5.5.4 Costs

There is no additional cost for the research subjects, nor for the institution, and the procedures to be evaluated will be performed according to the routine and individual medical indication.

6. STUDY DRUG

6.1 Description of the study drug

Chloroquine is indicated for the prophylaxis and treatment of acute cases of malaria caused by *Plasmodium vivax*, *P. ovale* and *P. malariae*. It is also indicated for the treatment of hepatic amebiasis and, together with other drugs, it has clinical efficacy in rheumatoid arthritis, systemic lupus erythematosus and discoid lupus, sarcoidosis and photosensitivity diseases such as *porphyria cutanea tarda* and severe polymorphic eruptions triggered by light (https://www.far.fiocruz.br/wp-content/uploads/2017/02/Cloroquina-ProfSaude.pdf).

Chloroquine binds moderately (60%) to plasma proteins and undergoes considerable biotransformation through the hepatic cytochrome P450 system into active metabolites, desethylchloroquine and bidesethylchloroquine. These metabolites can reach plasma concentrations of 40 to 10% of the chloroquine concentration, respectively. The chloroquine S (+) enantiomer has both greater binding to plasma proteins and greater metabolic clearance than the R (+) enantiomer. The 4-aminoquinolines derivatives bind to nucleoproteins and interfere with protein synthesis; inhibit DNA and RNA polymerase. They concentrate on the parasite's digestive vacuoles, increasing the pH, interfering with the parasite's ability to metabolize and use red blood cell hemoglobin.

Chloroquine is well-absorbed in the gastrointestinal tract. Drug distribution is relatively slow in an apparently very large volume (>100 L/kg). Chloroquine binds moderately (60%) to plasma proteins and undergoes considerable biotransformation through liver CYP enzymes. Renal chloroquine clearance accounts for about half of its total systemic clearance.

Although the drug ideally has an injectable presentation in some countries, for this type of use in critically ill patients, it is not available in Brazil and in most countries.

Chloroquine exhibits complex pharmacokinetics in adults and children, so that plasma levels of the drug shortly after its administration are determined by the rate of distribution and not by the rate of elimination. Due to the extensive connection with the tissues, a loading dose

is necessary to obtain effective plasma concentrations. Chloroquine's half-life increases from (72 to 120) hours, as plasma levels decline. The terminal half-life varies from (30 to 60) days and traces of the drug may be found in the urine for years after therapeutic use. In addition to its antiparasitic effect, chloroquine has antipyretic and anti-inflammatory action. It presents fast and almost complete absorption; with plasma peak in (1 to 2) hours; It is widely distributed in tissues, concentrating on the iris, choroid and less on the cornea, retina, sclera and also on red blood cells. Chloroquine crosses the placenta and is distributed in breast milk. Plasma half-life of (72 to 120) hours; metabolized in the liver and excreted slowly in the urine. The chloroquine (chloroquine diphosphate) produced by Farmanguinhos is available in the form of a white circular, flat, grooved tablet.

6.2 Dosage and administration

Below is a description of the dosages for the groups in this study:

**Group 1 (higher dose – 10 days)**		
	**1st daily dose**	**2nd daily dose**
**D1 – D10**	**4 tablets of 150mg (600mg)**	**4 tablets of 150mg (600mg)**
**Group 2 (lower dose – 5 days CQ + 5 days placebo)**		
**D1 (Loading dose)**	**3 tablets of 150mg (450mg) + 1 tablet of placebo**	**3 tablets of 150mg (450mg) + 1 tablet of placebo**
**D2 – D5**	**3 tablets of 150mg + 1 tablets of placebo**	**4 tablets of placebo**
**D6 – D10**	**4 tablets of placebo**	**4 tablets of placebo**

The medication will be administered via oral administration (or via nasogastric tube in case of orotracheal intubation - OTI). All treatment medications will be provided by Farmanguinhos.

6.3 Dose escalation

Not applicable, as this would greatly increase the sample size, not allowing conclusions in time for its use in the current public emergency situation.

6.4 Dosage modifications and concomitant drugs

To be judged by the results that will be portrayed in the literature, in real time, the study's monitoring committee may decide to change the dosage used in the study, as well as the possibility of adding other concomitant drugs.

6.5 Acquisition

Therapeutic agents will be sent by Farmanguinhos to the referral hospitals.

6.6 Preparation, handling, storage and responsibilities

After the inclusion of the participants with suspected COVID-19, who are hospitalized in the participating hospital, the participants will receive a prescription for Chloroquine/Placebo and will be referred to the study pharmacist who will be responsible for randomization in the treatment group and allocation of study medications (methodology best described posteriorly).

All test drugs (chloroquine and placebo), will be from the same batch, stored in their original packaging in the hospital's clinical pharmacy sector. Chloroquine diphosphate/placebo tablets will be suitable for unit dose dispensing for each participant during the treatment period. A label with the study reference, patient number (to be filled in) and the information “Product for use in clinical studies” will be attached to each blister/package.

The investigational products will be stored in accordance with the manufacturer's recommendations, inside the original packaging and in places with restricted access; and in accordance with Good Drug Storage Practices, as defined by law.

The investigator or other team member with permission to store and distribute the experimental product is responsible for ensuring that the experimental product used in the clinical study is kept safe and in compliance with applicable regulatory requirements. Every experimental product must be distributed according to the randomization list and by the study

pharmacist to ensure that an accurate record of the distributed and returned experimental product is maintained. A record of all study medication will be kept in the investigator's file.

Data regarding the date of receipt, the batch number, the expiration date and the date of use will be recorded for each of the study medications. An experimental drug registration diary will be kept at the study clinic. Any question related to the quality perceived at the time of receipt or during the use of an experimental product (deficiency of the investigational product in terms of condition, appearance, relevant documentation, labeling, expiration date, etc.) must be notified immediately. Under no circumstances should the investigator supply the experimental product to third parties, allow the experimental product to be used for purposes other than those indicated by this clinical study protocol, or discard the experimental product in any other way.

6.7 Collection and/or destruction of medicines

It is the sponsor's responsibility to ensure the destruction of any partially used or unused Experimental Product. A detailed form describing the returned Experimental Product will be developed with the Investigator, and will be signed by the Investigator and the Monitoring Team.

6.8 Accounting and adherence to treatment

Each dose of study treatment will be administered by a study pharmacist, with the patient being monitored for 30 minutes after administration. If the patient vomits or rejects treatment during the 30 minutes, the same dose will be administered again. The study pharmacist should record the information on the appropriate CRF page. Next, the monitor responsible for the study will check the data in the CRF by comparing it with the Experimental Product he/she has collected and the treatment registration forms. All measures will be taken to ensure that patients receive treatment in accordance with the protocol.

7. MEASURES TO MINIMIZE BIAS: RANDOMIZATION AND BLINDING

An electronically generated randomization list was prepared by an independent statistician, with 110 blocks of 4 participants per block. This randomization list was generated on R software (Package 'blockrand'). The list will be accessible only to non-blinded pharmacists in the study, in an attempt to minimize observation bias. Participants will be randomized by the study pharmacist to their designated treatment regimen at the time of inclusion and will be subsequently identified throughout the study only by their allocated study number, always assigned following chronological order. In case of SAEs, unmasking will be available to DSMB members, and preliminary analyses will be performed even before the scheduled interim analyses, in order to guide early halting of any of the CQ arms. The randomization and blinding procedure will be described in a specific SOP.

7.1 Compliance with the study intervention

Each dose of the study product will be administered by a qualified and licensed member to administer the study product. Administration date and time will be inserted in the case report form (CRF).

7.2 Concomitant therapy

All concomitant therapies will have a medical indication, and will be registered in CRF, for later analysis.

7.3 Salvage drug

N/A

8. DISCONTINUATION OF THE INTERVENTION AND WITHDRAWAL OF THE STUDY

8.1 Criteria for stopping and discontinuing study intervention

8.1.1 Interruption of administration

The study drug will be maintained for 10 days according to the indicated group, regardless of their hospitalization for the same or longer period. Patients who are discharged before then will be contacted on the evaluation days provided for in the protocol.

8.1.2 Interruption of study for safety reasons

Given the severity of COVID-19 disease, there are no pre-specified stopping rules. Thus, there will be careful supervision by the protocol team and security monitoring committee, and frequent analyses, in real time.

8.1.3 Withdrawal from randomized treatment or study

Patients may withdraw from the study at any time, upon request, without any consequences. Patients should be listed as having their consent withdrawn only when they no longer wish to participate in the study and no longer authorize investigators to make efforts to continue obtaining information.

Efforts must be made to maintain patients entering and participating in the study. Patients should be told about the scientific importance of their data, even if they discontinue treatment with the study drug.

8.1.4 Study drug discontinuation

The patient can stop taking the study drug for any of the following reasons;

1. Patient requests to discontinue study medication;
2. The occurrence of any medical condition or circumstance that exposes the patient to substantial risk;

3. Any serious adverse event (SAE), clinically significant adverse event, serious laboratory complications, abnormality, intercurrent illness or other medical condition that suggests to the investigator that participation is not safe;

The reason for the patient discontinuing use of the study drug should be documented on the case report form

8.1.5 Withdrawal of patients from the study

A patient can be withdrawn from the study after initial administration for the following reasons:

1. The patient withdraws consent or requests to discontinue the study for any reason;
2. Death of the patient;
3. Study closure.

Patients who withdrew from this study after signing the consent and administration of the study product, will not be replaced. The reason for discontinuing the study patient will be recorded on the case report form.

8.1.6 Study interruption

1. If information about the product raises doubts about the risk-benefit ratio;
2. If the results of the clinical study do not appear to be scientifically convincing;
3. If there is no longer any interest in the clinical study arm or in the case of its obsolescence;
4. In case of non-compliance, by the researcher, with a fundamental obligation, including, among others, the violation of the clinical study protocol, the non-compliance with applicable laws and regulations or non-compliance with the ICH standards related to Good Clinical Practices;
5. If the treatment is instituted as standard treatment by the Ministry of Health or another reliable scientific society, national or international.

9. STUDY EVALUATIONS AND PROCEDURES

9.1 Definition of source documents

All data and information requested in the clinical record form (CRF) will be collected in the electronic medical record of the patients or in interviews with the patient or his caregiver, including vital and clinical signs, concomitant diseases and any possible concomitant medications. Demographic data will be recorded in the center file or in the patient's medical file (MedView electronic medical record system), together with the viremia results for COVID-19. **Clinical information and the results of laboratory tests will be obtained from the electronic hospital record and inputed by the team on tablets, which will have an online connection with REDCap, where the information will be stored in real time, using the FMT-HVD server.**

9.2 Security instructions

The safety and tolerability of treatments will be assessed based on the registration of Adverse Events (AEs) and the classification of laboratory assessments and vital signs. A severity rating scale, based on the toxicity rating scales developed by the WHO and the National Institutes of Health, Division of Microbiology and Infectious Diseases, will be used to rate the severity of all symptoms, physical examination findings and results of hemoglobin. All laboratory abnormalities - with Grade 2 or greater for biological data - with clinically significant abnormalities (regardless of degree) must be monitored until resolution or until their progression stabilizes.

Virology exams will be carried out at LACEN - AM, linked to the Health Surveillance Foundation, in partnership with the Leônidas & Maria Deane Institute. The coronavirus diagnostic protocols will follow the routine recommended by the Ministry of Health, with RT-PCR.

9.5 Monitoring of adverse events

All events must be controlled and reported in accordance with all applicable regulations and included in the final clinical study report. All physical/clinical symptoms described at each

visit will be recorded as an adverse event if they occur or worsen during the study and are considered to be related to the study drug.

9.6 Adverse Event (AE) and Serious Adverse Event (SAE) definitions

An AE is any unwanted medical occurrence in a patient or clinical study participant who is administered a pharmaceutical product, and this occurrence does not necessarily have to be causally related to treatment. A priori, the efficacy outcomes specified in the protocol will not be considered as AEs, unless, due to the evolution, severity or any other characteristics of these events, the Investigator, in his clinical judgment, considers them exceptional for the situation in question. question. A Severe AE is any unwanted medical occurrence that, in any dose:

1. Result in death or endangers life;
2. Demand the patient's hospitalization or the extending of an existing hospitalization;
3. Results in disability or significant/persistent incapacity;
4. Birth defect or congenital anomaly;
5. Constitutes a clinically important event.

Based on a medical and scientific analysis, any other situations in which an urgent report would be appropriate, such as important medical events that may not immediately threaten life or result in death or hospitalization, but that may leave the patient vulnerable or require intervention to avoid one of the other results listed in the definition above.

10. STATISTICS

10.1 Analytical plan

Descriptive statistics will be used for demographic, laboratory and clinical data. To assess the safety of the high and the low dosages of CQ the proportion (95% CI) of deaths in each group will be compared with the historical proportion (95% CI) of deaths in patients who did not use CQ in other countries. For qualitative variables, Chi-square tests and Fisher's exact test will be performed. t-test or Mann-Whitney test will be used for means/median comparisons. An cumulative proportion of deaths will be assessed by survival models, using Kaplan-Meier estimate curves. Log-rank and Peto-Peto (correction for low observation numbers in the end of the follow-up) tests will be used for survival time to event analyses.

Statistical analyses will be performed in the R statistical package (v3.6.1), and $p<0.05$ will be considered significant.

10.2 Sample calculation and interim analysis

The sample for the primary outcome (reduction in lethality in patients with severe respiratory virus treated with a high dosage of chloroquine compared to a low dosage) was calculated based on the following assumptions:

3. The 20% lethality incidence in critically ill patients per year in the low dosage group;

4. High dosage chloroquine will be able to reduce lethality by at least 50% compared to low dosage chloroquine;

Thus, considering a test of differences in proportions between 2 groups of the same size, with power of 80% and alpha of 5%, 394 participants will be needed (197 per group). Considering 10% losses, the sample size is 440 participants.

To include interim analyses, we recalculate the sample sizes required in each of the analyzes and in the final analysis, using an alpha-spending function approach, in which the type 1 error is controlled by alpha correction final, based on the alphas used for interim analyses. There are several alpha spending functions and there is always a trade-off between how much will be 'spent' on the interim analysis and the final sample size required. In summary, the more difficult it is to reject the null hypothesis in the interim analysis, the less alpha will be spent and the impact will not be large. We will use Hwang-Shih-DeCani spending functions with $\gamma = -4$ for the upper limit (that is, to interrupt the study that rejects the null hypothesis in the interim analysis), which corresponds to a conservative method, equivalent to the upper limit of O 'Brien-Fleming, with little impact on the final sample size. We will also include a lower limit for testing futility, also using the Hwang-Shih-DeCani spending function, but with a less stringent $\gamma = -2$ parameter for a beta spending function. Although the calculation includes a possible interruption due to futility, this can be ignored, at the discretion of the DSMB (or equivalent) without any impact on the type 1 error.

The table below shows the calculations for the 3 interim analyses and for the final analysis, showing both the sample size required in each analysis as well as the upper (one-tailed 2.5%) and lower limits.

		Inferior Limit		Superior Limit	
Sample size percentage	N	Z	p one-tailed	Z	p one-tailed
	10	-	0.278	3.1	
25%	8	0.59	7	6	0.0008
	21	0.3	0.638	2.8	
50%	5	5	6	2	0.0024
	32	1.1	0.882	2.4	
75%	2	9	1	4	0.0074
	42	2.0	0.978	2.0	
100%	9	1	0	1	0.0220

The procedure will be based on the calculation of the Z value of the difference of proportions test in each of the analyses, and the study will continue if this value is between the values of the lower and upper limits and will be interrupted if it is above the upper limit (by rejection H0) or below the lower limit (for futility), remembering that the latter can be ignored without prejudice to the calculations.

All calculations in this report were made in the R environment, version 3.6.1, with the functions implemented in the TrialSize and gsDesign packages. Other intermediary analyzes can be carried out throughout the study to periodically monitor the safety of participants and will provide possible prior information on treatment to the DSMB and Ministry of Health.

11. SUPPORTING DOCUMENTATION AND OPERATIONAL CONSIDERATIONS

11.1 Regulatory, ethical and supervisory considerations of the study

This study will be conducted in accordance with the principles set out in The Belmont Report: Ethical Principles and Guidelines for the Protection of Human Research and ICH E6 (R2).

The protocol will be submitted to CONEP, via Plataforma Brasil, in accordance with the regulations established by CONEP under COVID-19.

11.2 Informed consent process

Informed consent is a process initiated before an individual agrees to participate in a study and continues throughout the individual's participation. Investigators or the designated research team will obtain informed consent from the research subject. Participants will receive a concise and focused presentation of key information about the clinical trial, verbally and with written consent. The main information about the study will be organized and presented in layman’s terms and language that makes it easier to understand why someone may or may not want to participate.

The Informed Consent Term (ICF) will be approved by CONEP and the subjects must read and review the consent. The subjects (or legally authorized representatives) must sign the ICF before starting any study procedures that are being done specifically for this study. Once signed, a copy of the ICF will be given to the study subject.

Stored samples will be labeled with bar codes to maintain confidentiality. Identifiable data can occur as needed; however, confidentiality will be maintained as described for this protocol and with the approval of CONEP.

The samples will not be used for commercial purposes. Although the results of any future research may be patentable or commercially profitable, the subjects will have no financial interest in any commercial development resulting from future research.

There are no direct benefits for the individual. No secondary research results will be entered in the subject's medical record. Incidental findings will not be shared with the subject, including medically actionable incidental findings, unless required by law.

Individuals can withdraw permission to use samples for secondary use at any time. He/she will need to contact the study site and the samples will be removed from the study's biorepository.

11.3 End and termination of study

This study may be terminated prematurely if there is sufficient reasonable cause, including but not limited to:

1. Determination of unexpected, significant or unacceptable risks to subjects;
2. Results of the interim analysis;
3. Insufficient compliance with protocol requirements;
4. Data that is not sufficiently complete and / or cannot be evaluated;
5. Regulatory authorities decisions

If the study ends prematurely, the site PI will promptly inform the study subjects and CONEP, as applicable. The PI will ensure adequate follow-up of subjects, as needed.

11.4 Confidentiality and privacy

Confidentiality must be strictly maintained by participating investigators, and their staff. This confidentiality is extended to cover information relating to individuals, results of biological sample tests and genetic tests and all other information generated during participation in the study.

Information about the study subjects will not be disclosed to any unauthorized third party.

The confidentiality of the subject's personal information will be maintained when the results of the study are published or discussed.

The study monitor, other authorized sponsor representatives, CONEP representatives and/or regulatory agencies can inspect all necessary documents and records maintained by the investigator, including, but not limited to, medical records (office, clinic or hospital) and

pharmacy records of subjects of this study. The clinical study team will allow access to these records.

All source records, including electronic data, will be stored in secure systems in accordance with institutional policies and federal regulations.

All study data and research samples that leave the study site (including any data transmission) will be identified only by a coded number linked to a subject through a code maintained at the clinical site. Names or IDs will not be released unless the security monitoring committee approves and aligns with consent or in accordance with the laws for the required reports.

11.5 Secondary use of samples and stored data

Secondary Research in Human Subjects is the reuse of identifiable data or bio-specimens collected from some other "primary" or "initial" activity, such as the data and samples collected in this protocol. Any use of samples or data for secondary research purposes, however, will be presented in a separate protocol for approval by CONEP.

Each sample will be identified only with a barcode and a unique tracking number to protect the confidentiality of the subject's personal information.

11.6 Clinical monitoring

Monitoring of the clinical site is carried out to ensure that the rights and well-being of individuals in the study are protected, that reported study data is accurate, complete and verifiable. Clinical Monitoring also ensures that the conduct of the study is in compliance with the currently approved protocol/amendment (s), ICH, GCP and the applicable regulations, requirements and sponsor's requirements. Clinical monitoring will also verify that any critical study procedures are completed following specific instructions in the protocols.

Details of clinical site monitoring are documented in a clinical monitoring plan (CMP). The CMP describes in detail who will carry out the monitoring, how often the monitoring will be carried out and the distribution of the monitoring reports.

Monitoring visits will include, but are not limited to, review of regulatory files, accountability records, CRFs, Informed Consent Forms, medical and laboratory reports, on-site study on intervention storage records, training records and protocol compliance and GCP.

Monitors will have access to each study site, participant, study personnel and all documentation in accordance with the monitoring plan (to be carried out after CONEP approval).

Study monitors will meet with investigators to discuss any outstanding issues and documentary issues.

11.7 Data management and record keeping

Data collection is the responsibility of the clinical study team under the supervision of the PI 1. The PI must keep the documentation complete and accurate.

Clinical research data and source documentation (including, but not limited to, AE/SAE, concomitant medications, medical history, physical assessments, laboratory data) will be entered by the clinical study location personnel on the CRFs.

The data system includes password protection and internal quality checks, such as automatic range checks, to identify data that appears to be inconsistent, incomplete or inaccurate.

Retention of study records and study-related records, including regulatory filing, drug registration, consent forms, source documents and electronic records must be kept for a period of 2 years after the study closure date. These documents must be retained for a longer period, however, if required by local authorities.

Data recorded in the CRF and derived documents must be consistent with the data recorded in the source document.

11.8 Protocol deviations

A protocol deviation is any non-compliance with the clinical trial protocol. Noncompliance can be on the part of the subject, the investigator or the team of the study site. After a deviation, corrective actions must be developed and implemented promptly. All individual protocol deviations will be addressed in the subject's study records.

It is the responsibility of the PI and site personnel to use continuous surveillance to identify and report deviations within five working days after the protocol deviation is identified.

All deviations must be reported immediately according to the protocol deviation reporting procedures.

Protocol deviations must be sent to CONEP, according to their guidelines.

11.9 Data publishing and sharing policy

To avoid premature release of data, this protocol specifies that the efficacy data for a study that has not yet been completed due to insufficient enrollment should not be published.

An independent monitoring committee will review the results of an interim analysis of study data to make recommendations on whether the study should continue or not.

It is important to note that investigators will remain blind to any analysis results; study data will only be released if the trial is interrupted based on a recommendation from the monitoring committee or has reached its target number of outcomes or amount of follow-up from participants.

11.10 Conflict of interest policy

The independence of this study from any real or perceived influence, such as by the pharmaceutical industry, is fundamental. Therefore, any real conflict of interest of people who have a role in the design, conduct, analysis, publication or any aspect of this test will be disclosed and managed.

11.11 Amendments to the clinical study protocol

All appendices to this document and those indicated here are part of this clinical study protocol. The investigator shall not modify or alter the clinical study protocol without prior analysis and CONEP's written approval/favorable opinion of an amendment, except when necessary to rule out immediate risk to patients in the clinical study, or when the change involves only logistical or administrative aspects of the study.

Any changes that are be agreed must be registered in writing, and the written amendment must be signed by the investigator and filed with this clinical study protocol. Any amendment to the clinical study protocol requires prior written approval/favorable opinion from CONEP, prior to its implementation, unless there are priority security reasons.

In some cases, an amendment may require a change to the Informed Consent Form. The researcher must receive approval / favorable opinion from CONEP, regarding the revised Free and Informed Consent Term, before the change is put into practice.

12. REFERENCES

1. Rothan HA, Byrareddy SN. The epidemiology and pathogenesis of coronavirus disease (COVID-19) outbreak. *J Autoimmun*. February 2020:102433. doi:10.1016/j.jaut.2020.102433.
2. Wang C, Horby PW, Hayden FG, Gao GF. A novel coronavirus outbreak of global health concern. *Lancet*. 2020;395(10223):470-473.

3. Ren L-L, Wang Y-M, Wu Z-Q, Xiang Z-C, Guo L, Xu T, Jiang Y-Z, Xiong Y, Li Y-J, Li X-W, Li H, Fan G-H, Gu X-Y, Xiao Y, Gao H, Xu J-Y, Yang F, Wang X-M, Wu C, Chen L, Liu Y-W, Liu B, Yang J, Wang X-R, Dong J, Li L, Huang C-L, Zhao J-P, Hu Y, Cheng Z-S, Liu L-L, Qian Z-H, Qin C, Jin Q, Cao B, Wang J-W. Identification of a novel coronavirus causing severe pneumonia in human. *Chin Med J (Engl)*. February 2020:1.

4. Lu H. Drug treatment options for the 2019-new coronavirus (2019-nCoV). *Biosci Trends*. 2020;14(1).

5. Wang W, Tang J, Wei F. Updated understanding of the outbreak of 2019 novel coronavirus (2019-nCoV) in Wuhan, China. *J Med Virol*. 2020;92(4):441-447. doi:10.1002/jmv.25689.

6. Bassetti M, Vena A, Giacobbe DR. The novel Chinese coronavirus (2019-nCoV) infections: Challenges for fighting the storm. *Eur J Clin Invest*. 2020;50(3). doi:10.1111/eci.13209.

7. Holshue ML, DeBolt C, Lindquist S, Lofy KH, Wiesman J, Bruce H, Spitters C, Ericson K, Wilkerson S, Tural A, Diaz G, Cohn A, Fox L, Patel A, Gerber SI, Kim L, Tong S, Lu X, Lindstrom S, Pallansch MA, Weldon WC, Biggs HM, Uyeki TM, Pillai SK. First Case of 2019 Novel Coronavirus in the United States. *N Engl J Med*. January 2020. doi:10.1056/nejmoa2001191.

8. Remuzzi A, Remuzzi G. Imported from https://www.thelancet.com/journals/lancet/article/PIIS0140-6736(20)30627-9/fulltext?utm_campaign=tlcoronavirus20&utm_source=twitter&utm_medium=social. *Lancet*. 0(0).

Bergeron E, Benjannet S, Erickson BR, Rollin PE, Ksiazek TG, Seidah NG, Nichol ST. Chloroquine is a potent inhibitor of SARS coronavirus infection and spread. *Virol J*. 2005;2. doi:10.1186/1743-422X-2-69.

10. Wang M, Cao R, Zhang L, Yang X, Liu J, Xu M, Shi Z, Hu Z, Zhong W, Xiao G. Remdesivir and chloroquine effectively inhibit the recently emerged novel coronavirus (2019-nCoV) in vitro. *Cell Res*. 2020;30(3). doi:10.1038/s41422-020-0282-0.

11. Touret F, de Lamballerie X. Of chloroquine and COVID-19. *Antiviral Res*. 2020;177. doi:10.1016/j.antiviral.2020.104762.

12. Gao J, Tian Z, Yang X. Breakthrough: Chloroquine phosphate has shown apparent efficacy in treatment of COVID-19 associated pneumonia in clinical studies. *Biosci Trends*. 2020;14(1). doi:10.5582/bst.2020.01047.

13. Colson P, Rolain JM, Raoult D. Chloroquine for the 2019 novel coronavirus SARS-CoV-2. *Int J Antimicrob Agents*. 2020;55(3). doi:10.1016/j.ijantimicag.2020.105923.

14. multicenter collaboration group of Department of Science and Technology of Guangdong Province and Health Commission of Guangdong Province for chloroquine in the treatment of novel coronavirus pneumonia. [Expert consensus on chloroquine phosphate for the treatment of novel coronavirus pneumonia]. *Zhonghua Jie He He Hu Xi Za Zhi*. 2020;43(0):E019. doi:10.3760/cma.j.issn.1001-0939.2020.0019.

15. Keyaerts E, Vijgen L, Maes P, Neyts J, Ranst M Van. In vitro inhibition of severe acute respiratory syndrome coronavirus by chloroquine. *Biochem Biophys Res Commun*. 2004;323(1):264-268. doi:10.1016/j.bbrc.2004.08.085.

16. Lim HS, Im JS, Cho JY, Bae KS, Klein TA, Yeom JS, Kim TS, Choi JS, Jang IJ, Park JW. Pharmacokinetics of hydroxychloroquine and its clinical implications in chemoprophylaxis against malaria caused by plasmodium vivax. *Antimicrob Agents Chemother*. 2009;53(4):1468-1475. doi:10.1128/AAC.00339-08.

17. Chloroquine Retinopathy - an overview | ScienceDirect Topics. https://www.sciencedirect.com/topics/medicine-and-dentistry/chloroquine-retinopathy. Accessed March 20, 2020.

18. Kazi MS, Saurabh K, Rishi P, Rishi E. Delayed onset chloroquine retinopathy presenting 10 years after long-term usage of chloroquine. *Middle East Afr J Ophthalmol*. 2013;20(1):89-91. doi:10.4103/0974-9233.106404.

19. Mavrikakis I, Sfikakis PP, Mavrikakis E, Rougas K, Nikolaou A, Kostopoulos C, Mavrikakis M. The incidence of irreversible retinal toxicity in patients treated with hydroxychloroquine: A reappraisal. *Ophthalmology*. 2003;110(7):1321-1326. doi:10.1016/S0161-6420(03)00409-3.

20. Inglot AD. Comparison of the antiviral activity in vitro of some non-steroidal anti-inflammatory drugs. *J Gen Virol*. 1969;4(2):203-214. doi:10.1099/0022-1317-4-2-203.

21. Miller DK, Lenard J. Antihistaminics, local anesthetics, and other amines as antiviral agents. *Proc Natl Acad Sci U S A*. 1981;78(6 I):3605-3609. doi:10.1073/pnas.78.6.3605.

22. Keyaerts E, Li S, Vijgen L, Rysman E, Verbeeck J, Van Ranst M, Maes P. Antiviral activity of chloroquine against human coronavirus OC43 infection in newborn mice. *Antimicrob Agents Chemother*. 2009;53(8):3416-3421. doi:10.1128/AAC.01509-08.

23. Tan YW, Yam WK, Sun J, Chu JJH. An evaluation of Chloroquine as a broad-acting antiviral against Hand, Foot and Mouth Disease. *Antiviral Res*. 2018;149:143-149. doi:10.1016/j.antiviral.2017.11.017.

24. Li C, Zhu X, Ji X, Quanquin N, Deng YQ, Tian M, Aliyari R, Zuo X, Yuan L, Afridi SK, Li XF, Jung JU, Nielsen-Saines K, Qin FXF, Qin CF, Xu Z, Cheng G. Chloroquine, a FDA-approved Drug, Prevents Zika Virus Infection and its Associated Congenital Microcephaly in Mice. *EBioMedicine*. 2017;24:189-194. doi:10.1016/j.ebiom.2017.09.034.

25. Yan Y, Zou Z, Sun Y, Li X, Xu KF, Wei Y, Jin N, Jiang C. Anti-malaria drug chloroquine is highly effective in treating avian influenza A H5N1 virus infection in an animal model. *Cell Res*. 2013;23(2):300-302. doi:10.1038/cr.2012.165.

26. Wang M, Cao R, Zhang L, Yang X, Liu J, Xu M, Shi Z, Hu Z, Zhong W, Xiao G. Remdesivir and chloroquine effectively inhibit the recently emerged novel coronavirus (2019-nCoV) in vitro. *Cell Res*. 2020;30(3):269-271. doi:10.1038/s41422-020-0282-0.

27. Gao J, Tian Z, Yang X. Breakthrough: Chloroquine phosphate has shown apparent efficacy in treatment of COVID-19 associated pneumonia in clinical studies. *Biosci Trends*. February 2020.

28. [Expert Consensus on Chloroquine Phosphate for the Treatment of Novel Coronavirus Pneumonia]. *Zhonghua Jie He He Hu Xi Za Zhi*. 2020;43(3). doi:10.3760/CMA.J.ISSN.1001-0939.2020.03.009.

29. Pagina niet gevonden | LCI richtlijnen. https://lci.rivm.nl/covid-19/bijlage/behandeladvies. (Accessed on 6th March 2020).
