# Supplementary material for: Effect of High vs Low Doses of Chloroquine Diphosphate as Adjunctive Therapy for Patients Hospitalized With Severe Acute Respiratory Syndrome Coronavirus 2 (SARS-CoV-2) Infection: A Randomized Clinical Trial
Source: JAMA Netw Open. 2020 Apr 24;3(4):e208857. doi: 10.1001/jamanetworkopen.2020.8857 (PMC12124691; doi:10.1001/jamanetworkopen.2020.8857)
Supplement: Supplement 2. — eTable. Clinical Details and Cumulative CQ Dosage per Kilogram in 12 Patients With QTcF Prolongation and Ventricular Tachycardia [file jamanetwopen-e208857-s002.pdf]

Supplementary Online Content

Borba MGS, Val FFA, Sampaio VS, et al; CloroCovid-19 Team. Effect of high vs low doses of chloroquine diphosphate as adjunctive therapy for patients hospitalized with severe acute respiratory syndrome coronavirus 2 (SARS-CoV-2) infection: a randomized clinical trial. *JAMA Netw Open*. 2020;3(4.23):e208857.
doi:10.1001/jamanetworkopen.2020.8857

eTable. Clinical Details and Cumulative CQ Dosage per Kilogram in 12 Patients With QTcF Prolongation and Ventricular Tachycardia

This supplementary material has been provided by the authors to give readers additional information about their work.

© 2020 Borba MGS et al. *JAMA Network Open.*

eTable 1. Clinical Details and Cumulative CQ Dosage per Kilogram in 12 Patients With QTcF Prolongation and Ventricular Tachycardia

**Age (years)**	**Gender**	**Ethnicity**	**First prolonged QTcF (ms)**	**Ventricular tachycardia**	**Comorbidities**	**Death**	**Cumulated CQ dosage until first prolonged QTcF (mg/kg)**	**Day of the first prolonged QTcF or VT**	**Day of death**	**Study arm**
40s	Male	Mixed	508	No	Obesity	Yes	32.1	2	6	High dosage§
40s	Male	Mixed	NA	Yes	Hypertension	Yes	14.1	1	1	High dosage§
60s	Male	Mixed	543	No	Hypertension, diabetes, and previous acute myocardial infarction	Yes	42.4	2	11	High dosage§
50s	Female	Mixed	507	Yes	Obesity and chronic kidney disease	Yes	56.5	3	6	High dosage§
60s	Female	Mixed	520	No	Hypertension, diabetes, HIV/Aids and chronic kidney disease	Yes	36.9	1	11	High dosage§
20s	Female	White	545	No	Hypertension and chronic kidney disease	No	48.8	4	-	Low Dosage¶
30s	Male	Mixed	502	No	Obesity	No	24.2	4	-	Low Dosage¶
60s	Female	Mixed	514	No	Hypertension and former smoking	No	51.4	2	-	High dosage§
80s	Male	Mixed	557	No	No comorbidities	Yes	48.0	1	8	High dosage§
30s	Female	Mixed	559	No	Rheumatic disease	No	45.0	4	-	Low Dosage¶
40s	Female	Mixed	502	No	Diabetes and liver disease	No	42.2	3	-	Low Dosage¶
60s	Male	Mixed	573	No	Cardiac bypass	Yes	30.0	1	2	High dosage§

¶ Low dose CQ for 5 days (450mg CQ twice daily on the first day and 450mg once a day for the remaining 4 days);
§ High dose CQ (600 mg CQ twice daily for 10 days).
NA: non-available

© 2020 Borba MGS et al. *JAMA Network Open.*
