# Supplementary material for: Effect of High vs Low Doses of Chloroquine Diphosphate as Adjunctive Therapy for Patients Hospitalized With Severe Acute Respiratory Syndrome Coronavirus 2 (SARS-CoV-2) Infection: A Randomized Clinical Trial
Source: JAMA Netw Open. 2020 Apr 24;3(4):e208857. doi: 10.1001/jamanetworkopen.2020.8857 (PMC12124691; doi:10.1001/jamanetworkopen.2020.8857)
Supplement: Supplement 3. — Data Sharing Statement [file jamanetwopen-e208857-s003.pdf]

Data Sharing Statement

Borba. Effect of High vs Low Doses of Chloroquine Diphosphate as Adjunctive Therapy on Patients Hospitalized With Severe Acute Respiratory Syndrome Coronavirus 2 (SARS-CoV-2) Infection. *JAMA Netw Open*. Published April 24, 2020.
10.1001/jamanetworkopen.2020.8857

Data
Data available: No
